# Effects of tranexamic acid on death, disability, vascular occlusive events and other morbidities in patients with acute traumatic brain injury (CRASH-3): a randomised, placebo-controlled trial

**DOI:** 10.1016/S0140-6736(19)32233-0

**Published:** 2019-11-09

**Authors:** The CRASH-3 trial collaborators

## Abstract

**Background:**

Tranexamic acid reduces surgical bleeding and decreases mortality in patients with traumatic extracranial bleeding. Intracranial bleeding is common after traumatic brain injury (TBI) and can cause brain herniation and death. We aimed to assess the effects of tranexamic acid in patients with TBI.

**Methods:**

This randomised, placebo-controlled trial was done in 175 hospitals in 29 countries. Adults with TBI who were within 3 h of injury, had a Glasgow Coma Scale (GCS) score of 12 or lower or any intracranial bleeding on CT scan, and no major extracranial bleeding were eligible. The time window for eligibility was originally 8 h but in 2016 the protocol was changed to limit recruitment to patients within 3 h of injury. This change was made blind to the trial data, in response to external evidence suggesting that delayed treatment is unlikely to be effective. We randomly assigned (1:1) patients to receive tranexamic acid (loading dose 1 g over 10 min then infusion of 1 g over 8 h) or matching placebo. Patients were assigned by selecting a numbered treatment pack from a box containing eight packs that were identical apart from the pack number. Patients, caregivers, and those assessing outcomes were masked to allocation. The primary outcome was head injury-related death in hospital within 28 days of injury in patients treated within 3 h of injury. We prespecified a sensitivity analysis that excluded patients with a GCS score of 3 and those with bilateral unreactive pupils at baseline. All analyses were done by intention to treat. This trial was registered with ISRCTN (ISRCTN15088122), ClinicalTrials.gov (NCT01402882), EudraCT (2011-003669-14), and the Pan African Clinical Trial Registry (PACTR20121000441277).

**Results:**

Between July 20, 2012, and Jan 31, 2019, we randomly allocated 12 737 patients with TBI to receive tranexamic acid (6406 [50·3%] or placebo [6331 [49·7%], of whom 9202 (72·2%) patients were treated within 3 h of injury. Among patients treated within 3 h of injury, the risk of head injury-related death was 18·5% in the tranexamic acid group versus 19·8% in the placebo group (855 *vs* 892 events; risk ratio [RR] 0·94 [95% CI 0·86–1·02]). In the prespecified sensitivity analysis that excluded patients with a GCS score of 3 or bilateral unreactive pupils at baseline, the risk of head injury-related death was 12·5% in the tranexamic acid group versus 14·0% in the placebo group (485 *vs* 525 events; RR 0·89 [95% CI 0·80–1·00]). The risk of head injury-related death reduced with tranexamic acid in patients with mild-to-moderate head injury (RR 0·78 [95% CI 0·64–0·95]) but not in patients with severe head injury (0·99 [95% CI 0·91–1·07]; p value for heterogeneity 0·030). Early treatment was more effective than was later treatment in patients with mild and moderate head injury (p=0·005) but time to treatment had no obvious effect in patients with severe head injury (p=0·73). The risk of vascular occlusive events was similar in the tranexamic acid and placebo groups (RR 0·98 (0·74–1·28). The risk of seizures was also similar between groups (1·09 [95% CI 0·90–1·33]).

**Interpretation:**

Our results show that tranexamic acid is safe in patients with TBI and that treatment within 3 h of injury reduces head injury-related death. Patients should be treated as soon as possible after injury.

**Funding:**

National Institute for Health Research Health Technology Assessment, JP Moulton Charitable Trust, Department of Health and Social Care, Department for International Development, Global Challenges Research Fund, Medical Research Council, and Wellcome Trust (Joint Global Health Trials scheme).

**Translations:**

For the Arabic, Chinese, French, Hindi, Japanese, Spanish and Urdu translations of the abstract see Supplementary Material.

## Introduction

Each year, worldwide, there are more than 60 million new cases of traumatic brain injury (TBI).^1^ Road traffic crashes and falls are the main causes and the incidence is increasing.^1^ Intracranial bleeding is a common complication of TBI and increases the risk of death and disability.^2^ Although bleeding can start from the moment of impact, it often continues for several hours after injury.^3^, ^4^ Ongoing intracranial bleeding can lead to raised intracranial pressure, brain herniation, and death. Tranexamic acid reduces bleeding by inhibiting the enzymatic breakdown of fibrin blood clots (fibrinolysis). The CRASH-2 trial^5^, ^6^ showed that in patients with trauma with major extracranial bleeding, early administration (within 3 h of injury) of tranexamic acid reduces bleeding deaths by a third. Subsequent analyses showed that even a short delay in treatment reduces the benefit of tranexamic acid administration.^7^ On the basis of these results, tranexamic acid was included in guidelines for the pre-hospital care of patients with trauma, although patients with isolated TBI were specifically excluded. However, increased fibrinolysis, as indicated by increased concentrations of fibrinogen degradation products, is often seen in patients with TBI and predicts intracranial haemorrhage expansion.^8^ Therefore, early administration of tranexamic acid in patients with TBI might prevent or reduce intracranial haemorrhage expansion and thus avert brain herniation and death.

**Research in context**

**Evidence before this study**

Evidence from the CRASH-2 trial that administration of tranexamic acid within 3 h of injury reduces death in patients with traumatic extracranial bleeding raised the possibility that tranexamic acid might reduce death from traumatic intracranial bleeding. Intracranial bleeding is common after traumatic brain injury (TBI) and increases head injury-related death and disability. Before the CRASH-3 trial, we made a systematic search for all randomised trials of tranexamic acid in acute traumatic injury. We searched PubMed, Science Citation Index, National Research Register, Zetoc, SIGLE, Global Health, LILACS, Current Controlled Trials, the Cochrane Injuries Group Specialised Register, CENTRAL, MEDLINE, and Embase for all publications until July 15, 2010. Details of our search were published previously. We found two small randomised trials of tranexamic acid in traumatic brain injury with a total of 510 patients. Meta-analysis of the two trials showed a statistically significant reduction in death with tranexamic acid. However, given the small size of the trials, we considered this evidence to be hypothesis generating, requiring confirmation in larger randomised trials.

**Added value of this study**

Our study found that the risk of death from head injury was reduced in patients treated with tranexamic acid, particularly when patients who had a Glasgow Coma Scale score of 3 and those with bilateral unreactive pupils at baseline were excluded. We found no evidence of any increase in disability among survivors. The risk of vascular occlusive events was similar in the tranexamic acid and placebo groups.

**Implications of all the available evidence**

On Aug 30, 2019, an updated search for randomised trials of the early administration of tranexamic acid in patients with traumatic brain injury identified one randomised trial in addition to the CRASH-3 trial. This study was a randomised trial of pre-hospital tranexamic acid in 967 patients with traumatic brain injury. The dose of tranexamic acid was the same as in the CRASH-3 trial and patients with a GCS score of 3 and those with unreactive pupils at baseline were also excluded. When the two trials were pooled, we found a reduction in head injury-related death with tranexamic acid and no evidence of an increased risk in vascular occlusive events or seizures. Combining the results of all available randomised trials shows a reduction in head injury-related death in patients treated with tranexamic acid. Early administration of tranexamic acid should be considered in patients with traumatic brain injury.

Before the CRASH-3 trial, only two small trials of tranexamic acid in patients with TBI had been done.^9^, ^10^ Meta-analysis of these trials showed a reduction in mortality with tranexamic acid (risk ratio [RR] 0·63 [95% CI 0·40–0·99]) but provided no evidence about the effect of tranexamic acid on disability or adverse events. The CRASH-3 trial aimed to quantify the effects of tranexamic acid on head injury-related death, disability, and adverse events in patients with TBI.^11^

## Methods

### Study design and participants

The CRASH-3 trial was an international, multi-centre, randomised, placebo-controlled trial of the effects of tranexamic acid on death and disability in patients with TBI. Adults with TBI who were within 3 h of injury, had a Glasgow Coma Scale (GCS) score of 12 or lower or any intracranial bleeding on CT scan, and no major extracranial bleeding were eligible. The fundamental eligibility criterion was that the responsible clinician was substantially uncertain as to the appropriateness of tranexamic acid treatment. The time window for eligibility was originally within 8 h of injury. However, on Sept 6, 2016, in response to evidence external to the trial indicating that tranexamic acid is unlikely to be effective when initiated beyond 3 h of injury,^6^, ^12^, ^13^ the trial steering committee amended the protocol to limit recruitment to within 3 h of injury and the primary endpoint was changed to head injury death in hospital within 28 days of injury for patients treated within 3 h of injury. This change was made without reference to the unblinded trial data. The data monitoring committee was not consulted about the change. The trial was done according to good clinical practice guidelines.

Because of the nature of their injury, most patients with TBI are unable to provide informed consent to participate in a clinical trial. As acknowledged in the Declaration of Helsinki, patients who are incapable of giving consent are an exception to the general rule of informed consent in clinical trials.^14^ In this trial, consent was usually sought from the patient's relative or a legal representative. If no such representative was available, the study proceeded with the agreement of two clinicians. If the patient regained capacity, he or she was told about the trial and written consent was sought to continue participation. If the patient or their representative declined consent, participation stopped. If patients were included in the trial but did not regain capacity, consent was sought from a relative or legal representative. We adhered to the requirements of the local and national ethics committees.

### Randomisation and masking

An independent statistician from Sealed Envelope (London, UK) prepared the randomisation codes and gave them to the drug packers so that treatment packs could be prepared. We randomly allocated eligible patients to receive tranexamic acid or matching placebo (0·9% sodium chloride) by intravenous infusion. After baseline information was collected on the entry form, the lowest numbered treatment pack remaining was taken from a box of eight treatment packs. At this point, provided that the ampoules inside the treatment pack were intact, the patient was considered to be randomised. Entry form data were entered into a secure online database by the trial investigators. Participants and study staff (site investigators and trial coordinating centre staff) were masked to allocation. An emergency unblinding service was available for use if the clinician believed that clinical management depended importantly on knowledge of whether the patient received tranexamic acid or placebo. The tranexamic acid was manufactured by Pfizer (Sandwich, UK). The Torbay and South Devon Healthcare NHS Trust (UK) prepared the 0·9% sodium chloride placebo. Ampoules and packaging were identical in appearance. The preparation of the treatment packs was done by Bilcare (Crickhowell, UK), by removal of the manufacturer's label and replacement with the trial label and treatment pack number. Pack label texts were identical for tranexamic acid and placebo. We checked the coding of the blinded ampoules by randomly testing each batch of treatments and doing high performance liquid chromatography to identify the contents.

### Procedures

Patients were randomly allocated to receive a loading dose of 1 g of tranexamic acid infused over 10 min, started immediately after randomisation, followed by an intravenous infusion of 1 g over 8 h, or matching placebo. Every patient was assigned a uniquely numbered treatment pack, which contained four ampoules of either tranexamic acid (500 mg) or placebo, one 100 mL bag of 0·9% sodium chloride (to use with the loading dose), a syringe and needle, stickers with the trial details and randomisation number (for attaching to infusion bags, forms, and the medical records), and instructions. We separately provided information for patients and representatives, consent forms, and data collection forms. The stickers, instructions, leaflets, and forms were in local languages. Once randomised, we collected outcome data even if the treatment was not given. Outcome data were collected 28 days after randomisation, at discharge from the randomising hospital, or at death (whichever was first). Because the trial was assessed as low risk (tranexamic acid is widely used and the trial was considered to have a low risk of bias), we used central trial monitoring and central statistical monitoring in conjunction with investigator training, meetings, and written guidance. Trial investigators and their institutions provided direct access to the source data for trial-related monitoring, audits, and regulatory inspections. We planned to monitor approximately 10% of patient records on site. However, after changing the primary outcome we expanded our monitoring plan to include patients enrolled within 3 h of injury who subsequently died. We monitored 2436 (19%) of 12 737 patient records onsite or remotely (using videocall or telephone), including 1161 (67%) of the patients who died from head injury (the primary outcome). The team of monitors worked alongside local trial teams to verify data from the source data, including pre-hospital ambulance cards, admission registers, emergency department notes, CT scans, surgery notes, death registers, and death certificates.

### Outcomes

The primary outcome was head injury-related death in hospital within 28 days of injury in patients randomly assigned within 3 h of injury. Because most patients with TBI with a GCS score of 3 and those with bilateral unreactive pupils have a very poor prognosis regardless of treatment, their inclusion in the trial might bias any treatment effect towards the null. We therefore prespecified a sensitivity analysis that excluded these patients.^11^ Cause of death was assessed by the responsible clinician. Secondary outcomes were early head injury-related death (within 24 h after injury), all-cause and cause-specific mortality, disability, vascular occlusive events (myocardial infarction, stroke, deep vein thrombosis, and pulmonary embolism), seizures, complications, neurosurgery, days in intensive care unit, and adverse events within 28 days of randomisation. A diagnosis of deep vein thrombosis or pulmonary embolism was recorded only if a positive result was found on imaging (eg, ultrasound) or at a post-mortem examination.

We originally estimated that a trial with approximately 10 000 patients would have 90% power (two-sided α=1%) to detect a 15% relative reduction (20–17%) in mortality.^15^ However, we changed the primary outcome to head injury-related death in hospital within 28 days of injury in patients randomly assigned within 3 h of injury and limited recruitment to within 3 h of injury. We then increased the sample size to 13 000 to have approximately 10 000 patients treated within 3 h of injury.^11^

### Statistical analysis

We published a statistical analysis plan before unblinding.^11^ The plan gave our reasons for limiting recruitment to within 3 h of injury and stated that outcomes for patients treated after 3 h of injury would be presented separately. All analyses were on an intention-to-treat basis. For each binary outcome, we calculated RRs and 95% CIs. We did a complete case analysis with no imputation for missing data. The safety of participants was overseen by an independent data monitoring committee, which reviewed four unblinded interim analyses.

We planned to report the effects of tranexamic acid on the primary outcome stratified by three baseline characteristics: severity of head injury, time to treatment, and age. Severity of head injury was assessed using the baseline GCS score—mild to moderate (GCS 9–15) and severe (GCS 3–8)—and by pupil reactivity. We also assessed the effect of severity in a regression analysis that included continuous terms for GCS and its square. We expected that any beneficial effect of tranexamic acid would vary by time to treatment with earlier treatment being most effective. We examined this hypothesis in a subgroup analysis of the effect of tranexamic acid according to the estimated time interval between injury and treatment (≤1, >1 to ≤3, >3 h). We prespecified that this analysis would include patients treated within and beyond 3 h of injury. Because TBI severity, systolic blood pressure, and age could confound the effect of time to treatment on treatment effectiveness, we planned to control for these variables in a multivariable model. Because fibrinolytic activation after TBI might increase with age, we examined the effect of tranexamic acid on head injury-related death stratified by age: 30 years or younger, 31–60 years, older than 60 years. For subgroup analyses, we report p values for the test for heterogeneity.

### Role of the funding source

The funder of the study had no role in study design, data collection, data analysis, data interpretation, or writing of the report. The corresponding authors had full access to all the data in the study and had final responsibility for the decision to submit for publication.

## Results

**Figure 1:**
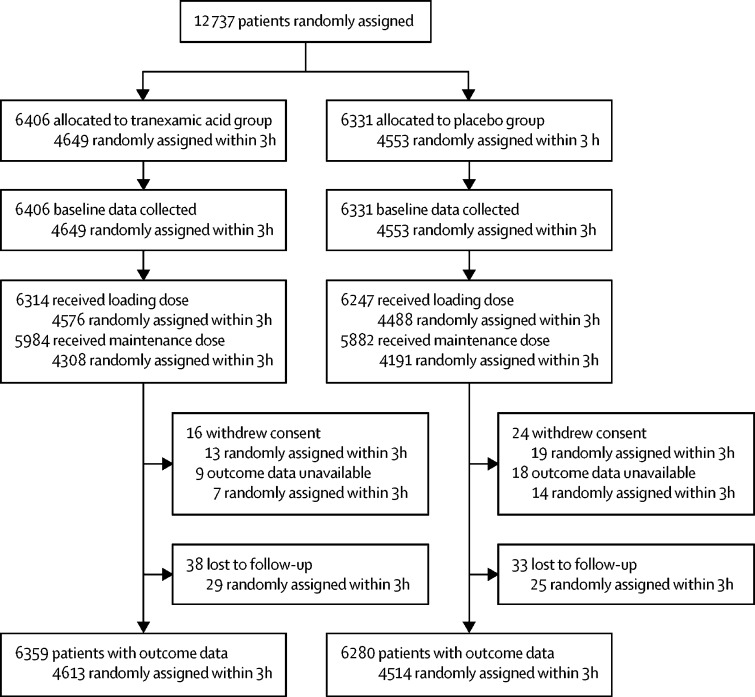
Trial profile

**Figure 2:**
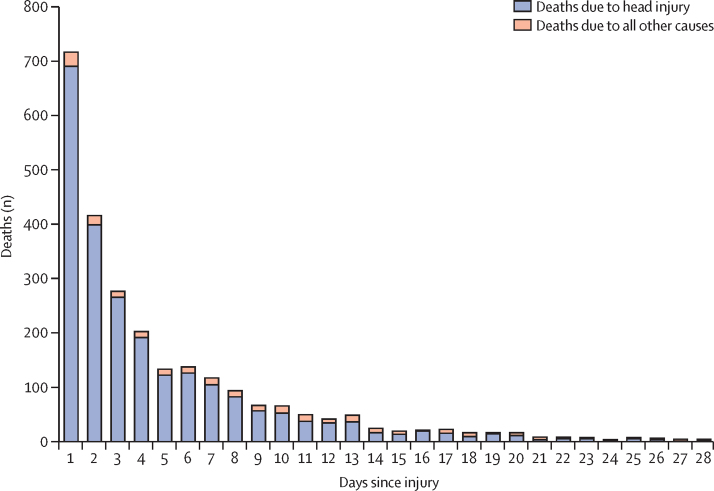
Mortality by days since injury among all patients

Between July 20, 2012, and Jan 31, 2019, we recruited patients with TBI from 175 hospitals in 29 countries. We stopped recruiting when the trial treatment expired. We randomly allocated 12 737 patients to receive tranexamic acid (6406 [50·3%]) or matching placebo (6331 [49·7%]), of whom 12 561 (98·6%) received the first dose of the allocated treatment (figure 1). We enrolled 9202 (72·2%) patients within 3 h of injury. 40 patients withdrew consent after randomisation but 13 of these agreed to outcome data collection or had outcome data collected as part of adverse event reporting. We did not obtain primary outcome data for 75 (0·8%) patients. There were 98 (0·8%) protocol violations. 66 (0·5%) patients did not meet the inclusion criteria (32 had GCS scores >12 and no bleeding on CT scan, 11 had major extracranial bleeding, eight had a time since injury >8 h, six were younger than 16 years, three had non-traumatic bleeding, five had a combination of the above reasons, and one patient received tranexamic acid before randomisation). 32 (0·3%) patients were recruited during a lapse in the annual renewal of ethics committee approval in the UAE. These patients were recruited according to the approved procedure and approval was reissued after the lapse. 13 patients were unmasked to treatment. Baseline characteristics were similar between treatment groups for patients treated within 3 h of injury (table 1) and for those treated after 3 h (appendix 8 p 1). Figure 2 shows the number of deaths due to head injury and all other causes by days since injury in all patients. 2560 deaths occurred and the median time to death was 59 h after injury (IQR 20–151).

**Table 1:** Baseline characteristics of patients before randomisation of those randomly assigned within 3 h of injury

	**Tranexamic acid (n=4649)**	**Placebo (n=4553)**
Sex*		
Men	3742 (80%)	3660 (80)
Women	906 (19%)	893 (20)
**Age, years**		
Mean (SD)	41·7 (19·0)	41·9 (19·0)
<25	1042 (22%)	996 (22%)
25–44	1716 (37%)	1672 (37%)
45–64	1169 (25%)	1184 (26%)
≥65	722 (16%)	701 (15%)
**Time since injury, h**		
Mean (SD)	1·9 (0·7)	1·9 (0·7)
≤1	877 (19%)	869 (19%)
>1–2	2003 (43%)	1889 (41%)
>2–3	1769 (38%)	1795 (39%)
**Systolic blood pressure, mm Hg**		
<90	89 (2%)	85 (2%)
90–119	1508 (32%)	1490 (33%)
120–139	1461 (31%)	1504 (33%)
≥140	1576 (34%)	1466 (32%)
Unknown	15 (<1%)	8 (<1%)
**Glasgow Coma Scale score**		
3	495 (11%)	506 (11%)
4	213 (5%)	213 (5%)
5	163 (4%)	172 (4%)
6	221 (5%)	232 (5%)
7	311 (7%)	294 (6%)
8	354 (8%)	315 (7%)
9	335 (7%)	292 (6%)
10	371 (8%)	364 (8%)
11	375 (8%)	390 (9%)
12	476 (10%)	478 (10%)
13	297 (6%)	312 (7%)
14	526 (11%)	458 (10%)
15	484 (10%)	492 (11%)
Unknown	28 (1%)	35 (1%)
**Pupil reaction**		
None reacted	425 (9%)	440 (10%)
One reacted	374 (8%)	353 (8%)
Both reacted	3706 (80%)	3636 (80%)
Unable to assess or unknown	144 (3%)	124 (3%)

Data are n (%), unless otherwise indicated.

* In the tranexamic acid group, one patient's sex was unknown.

Table 2 shows the effect of tranexamic acid on head injury-related death in the 9127 patients randomly assigned within 3 h of injury with outcome data. Among these patients, the risk of head injury-related death was 18·5% in the tranexamic acid group versus 19·8% in the placebo group (855 *vs* 892 events, RR 0·94 [95% CI 0·86–1·02]). In the prespecified sensitivity analysis that excluded patients with a GCS score of 3 or bilateral unreactive pupils at baseline, the results were 12·5% in the tranexamic acid group versus 14·0% in the placebo group (485 *vs* 525 events, 0·89 [0·80–1·00]).

**Table 2:** Effect of tranexamic acid on head injury-related death in patients randomly assigned within 3 h of injury

	**Tranexamic acid**	**Placebo**	**Risk ratio (95% CI)**
All	855/4613 (18·5%)	892/4514 (19·8%)	0·94 (0·86–1·02)
Excluding patients with GCS score of 3 or bilateral unreactive pupils*	485/3880 (12·5%)	525/3757 (14·0%)	0·89 (0·80–1·00)

GCS=Glasgow Coma Scale.

* Prespecified sensitivity analysis.

**Figure 3:**
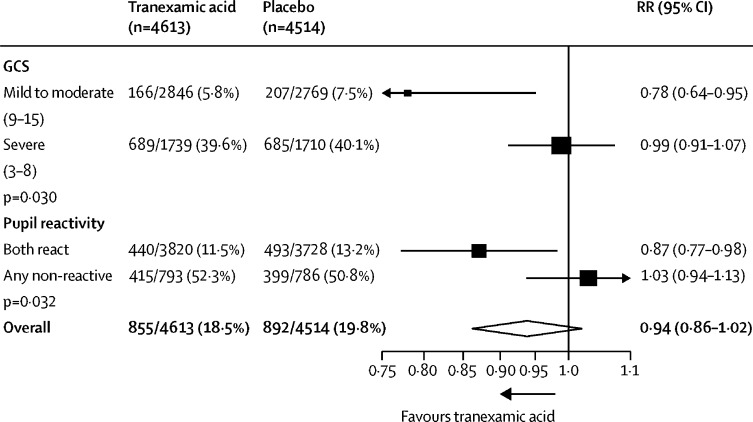
Effect of tranexamic acid on head injury-related death stratified by baseline severity in patients randomised within 3 h of injury RR=risk ratio. GCS=Glasgow Coma Scale.

We examined the effect of tranexamic acid on head injury-related death stratified by baseline GCS and pupillary reactions (figure 3). We found a reduction in the risk of head injury-related death with tranexamic acid in patients with mild-to-moderate head injury (RR 0·78 [95% CI 0·64–0·95]) but in patients with severe head injury (0·99 [0·91–1·07]) we found no clear evidence of a reduction (p value for heterogeneity 0·030). When we examined the effect of baseline GCS in a regression analysis we found evidence that tranexamic acid is more effective in less severely injured patients (p=0·007). Among patients with reactive pupils, head injury-related deaths were reduced with tranexamic acid (0·87, [0·77–0·98]).

**Figure 4:**
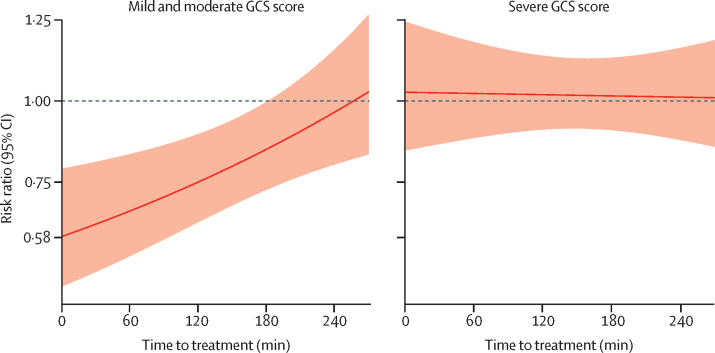
Effect of tranexamic acid on head injury-related death by severity and time to treatment in all patients The models were adjusted for GCS score, age, and systolic blood pressure. 537 patients with mild and moderate GCS scores (9–15) and 918 patients with severe GCS scores (4–8), excluding those with a GCS score of 3 and those with no reactive pupils, died because of head injury. GCS=Glasgow Coma Scale.

We examined the effect of tranexamic acid on head injury-related death stratified by time to treatment and recorded no evidence of heterogeneity (p=0·96). The RR of head injury-related death with tranexamic acid was 0·96 (95% CI 0·79–1·17) in patients randomly assigned within 1 h of injury, 0·93 (0·85–1·02) in those randomly assigned within more than 1 h and 3 h or fewer after injury, and 0·94 (0·81–1·09) in those randomly assigned more than 3 h after injury. However, as anticipated in the statistical analysis plan, patients who are treated soon after injury often have more severe head injury and so the effect of time to treatment could be confounded by severity. Figure 4 shows effect of time to treatment on the effect of tranexamic acid in patients with a mild and moderate head injury and in those with severe head injury after adjusting for GCS, systolic blood pressure, and age in a multivariable model including all participants. Early treatment was more effective than later treatment in patients with mild and moderate head injury (p=0·005) but we found no obvious effect of time to treatment in patients with severe head injury (p=0·73). The effectiveness of tranexamic acid by time to treatment stratified by severity is further shown in the appendix 8 (p 3). We found no evidence of heterogeneity in the effect of tranexamic acid by patient age (p=0·45).

We examined the effect of tranexamic acid on head injury-related death stratified by World Bank income group (high-income *vs* low-income and middle-income countries). This analysis was not prespecified. Although the reduction in the risk of head injury-related death with tranexamic acid was larger in high-income countries (RR 0·76 [95% CI 0·55–1·04]) than in low-income and middle-income countries (0·92 [0·81–1·04]), we found no statistical evidence of heterogeneity by country income group (p=0·26).

Because early head injury-related deaths are more likely to result from intracranial haemorrhage than are late head injury-related deaths, we examined the effect of tranexamic acid on head injury-related death within 24 h of injury. The RR of head injury-related death was 0·81 (95% CI 0·69–0·95) within 24 h. When patients with a GCS score of 3 and those with bilateral unreactive pupils at baseline were excluded, the RR was 0·72 (0·56–0·92) within 24 h.

The RR for non-head injury-related deaths was 1·31 (95% CI 0·93–1·85) and 0·96 (0·89–1·04) for all-cause mortality. The results for non-head injury-related deaths broken down by cause are presented in the appendix 8 (p 2).

We assessed the effect of tranexamic acid on disability in survivors by comparing the mean Disability Rating Scale score (lower score means less disabled) between the tranexamic acid and placebo groups. The mean scores were similar between groups for patients treated within 3 h of injury (4·99 [SD 7·6] in the tranexamic acid group *vs* 5·03 [7·6] in the placebo group) and for those treated after 3 h (4·52 [7·0] in the tranexamic acid group *vs* 5·00 [7·4] in the placebo group). We also examined the effect of tranexamic acid on disability (table 3) using an outcome measure designed by patient representatives by estimating the RR of being in the most extreme category for six areas of functioning (walking, washing, pain and discomfort, anxiety or depression, agitation or aggression, and fatigue). The prevalence of disability among survivors was similar between groups.

**Table 3:** Effect of tranexamic acid on disability, vascular occlusive events, and other complications in patients randomly assigned within 3 h, patients randomised beyond 3 h, and all patients

	**<3 h**			**≥3 h**			**All**		
	Tranexamic acid (n=4613)	Placebo (n=4514)	RR (95% CI)	Tranexamic acid (n=1746)	Placebo (n=1766)	RR (95% CI)	Tranexamic acid (n=6359)	Placebo (n=6280)	RR (95% CI)
Patient-derived disability measures*									
Confined to bed	579 (12·6%)	549 (12·2%)	1·03 (0·93–1·15)	190 (10·9%)	222 (12·6%)	0·87 (0·72–1·04)	769 (12·1%)	771 (12·3%)	0·99 (0·90–1·08)
Unable to wash or dress	580 (12·6%)	583 (12·9%)	0·97 (0·87–1·08)	195 (11·2%)	228 (12·9%)	0·87 (0·72–1·04)	775 (12·2%)	811 (12·9%)	0·94 (0·86–1·03)
Severe pain or discomfort	38 (0·8%)	29 (0·6%)	1·28 (0·79–2·08)	10 (0·6%)	10 (0·6%)	1·01 (0·42–2·42)	48 (0·8%)	39 (0·6%)	1·22 (0·80–1·85)
Severe anxiety or depression	43 (0·9%)	41 (0·9%)	1·03 (0·67–1·57)	19 (1·1%)	20 (1·1%)	0·96 (0·51–1·79)	62 (1·0%)	61 (1·0%)	1·00 (0·71–1·43)
Severe agitation or aggression	53 (1·1%)	53 (1·2%)	0·98 (0·67–1·43)	14 (0·8%)	27 (1·5%)	0·52 (0·28–1·00)	67 (1·1%)	80 (1·3%)	0·83 (0·60–1·14)
Severe fatigue	100 (2·2%)	101 (2·2%)	0·97 (0·74–1·27)	40 (2·3%)	43 (2·4%)	0·94 (0·61–1·44)	140 (2·2%)	144 (2·3%)	0·96 (0·76–1·21)
**Complications**†									
All vascular occlusive events	69 (1·5%)	60 (1·3%)	1·13 (0·80–1·59)	32 (1·8%)	42 (2·4%)	0·77 (0·49–1·21)	101 (1·6%)	102 (1·6%)	0·98 (0·74–1·28)
Pulmonary embolism	18 (0·4%)	18 (0·4%)	0·98 (0·51–1·88)	6 (0·3%)	14 (0·8%)	0·43 (0·17–1·13)	24 (0·4%)	32 (0·5%)	0·74 (0·44–1·26)
Deep vein thrombosis	15 (0·3%)	12 (0·3%)	1·22 (0·57–2·61)	4 (0·2%)	4 (0·2%)	1·01 (0·25–4·04)	19 (0·3%)	16 (0·3%)	1·17 (0·60–2·28)
Stroke	29 (0·6%)	23 (0·5%)	1·23 (0·71–2·13)	17 (1·0%)	19 (1·1%)	0·90 (0·47–1·74)	46 (0·7%)	42 (0·7%)	1·08 (0·71–1·64)
Myocardial infarction	9 (0·2%)	12 (0·3%)	0·73 (0·31–1·74)	9 (0·5%)	8 (0·5%)	1·14 (0·44–2·94)	18 (0·3%)	20 (0·3%)	0·89 (0·47–1·68)
Renal failure	73 (1·6%)	56 (1·2%)	1·28 (0·90–1·80)	27 (1·5%)	28 (1·6%)	0·98 (0·58–1·65)	100 (1·6%)	84 (1·3%)	1·18 (0·88–1·57)
Sepsis	297 (6·4%)	279 (6·2%)	1·04 (0·89–1·22)	114 (6·5%)	133 (7·5%)	0·87 (0·68–1·10)	411 (6·5%)	412 (6·6%)	0·99 (0·86–1·12)
Seizure	130 (2·8%)	105 (2·3%)	1·21 (0·94–1·56)	76 (4·4%)	81 (4·6%)	0·95 (0·70–1·29)	206 (3·2%)	186 (3·0%)	1·09 (0·90–1·33)
Gastrointestinal bleeding	16 (0·3%)	22 (0·5%)	0·71 (0·37–1·35)	8 (0·5%)	13 (0·7%)	0·62 (0·26–1·50)	24 (0·4%)	35 (0·6%)	0·68 (0·40–1·14)

RR=risk ratio.

* Includes survivors only.

† Includes fatal and non-fatal events.

The risk of vascular occlusive events and other complications was similar in the tranexamic acid and placebo groups (table 3). We found no evidence that tranexamic acid increased fatal or non-fatal stroke (RR 1·08 [95% CI 0·71–1·64]). The risk of seizures was similar between groups (1·09 [95% CI 0·90–1·33]). The numbers of other adverse events were similar between groups (appendix 8 p 4).

## Discussion

This trial provides evidence that the administration of tranexamic acid to patients with TBI within 3 h of injury reduces head injury-related death, with no evidence of adverse effects or complications. We found a substantial reduction in head injury-related deaths with tranexamic acid in patients with mild and moderate head injuries but no apparent reduction in those with severe head injury. We found no increase in disability among survivors.

**Figure 5:**
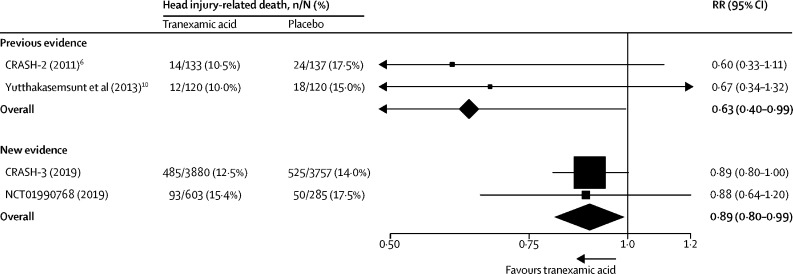
Evidence on the effect of tranexamic acid on head injury-related death RR=risk ratio.

Our trial had several strengths but also some limitations. The method of randomisation ensured that participating clinicians had no foreknowledge of the treatment allocation and the use of placebo control ensured that outcome assessments were blind to the intervention. Although the eligibility criteria required the recruiting clinician to be uncertain as to the appropriateness of tranexamic acid treatment, because tranexamic acid is not a recommended treatment for patients with isolated TBI, almost all patients with TBI who met the inclusion criteria were recruited. Baseline prognostic factors were well balanced and because almost all randomly assigned patients were followed up there was little potential for bias. The analysis was by intention to treat. The primary outcome was head injury-related death as assessed by the responsible clinician. Although some misclassification of cause of death is inevitable, the assessment was made blind to the trial treatment. All-cause mortality combines causes of death that might be affected by tranexamic acid (eg, head injury-related death due to intracranial bleeding) with causes that we do not expect to be affected by tranexamic acid (eg, sepsis) and therefore would be biased towards the null. Although the CRASH-3 trial is one of the largest trials in patients with TBI, the CIs were wide and compatible with a substantial reduction in head injury-related death and little or no benefit. On the other hand, when set in the context of all the available randomised trials of tranexamic acid in patients with TBI (figure 5), the possibility of no mortality benefit appears remote (NCT01990768).^9^, ^10^ When assessing outcome measures in clinical trials, provided there are few false positives (high specificity), estimates of the RR are unbiased even when sensitivity is imperfect.^16^ For this reason, a diagnosis of deep vein thrombosis or pulmonary embolism was recorded only if we found a positive result on imaging (eg, ultrasound) or at post-mortem examination. As a result, although the trial might have underestimated the risk of deep vein thrombosis or pulmonary embolism, the RR estimates for this outcome should be unbiased.

We anticipated that patients with TBI with a GCS score of 3 and those with bilateral unreactive pupils before treatment would have little potential to benefit from tranexamic acid and that their inclusion in the analysis would bias the treatment effect towards the null. Most patients with bilateral unreactive pupils already have extensive intracranial haemorrhage and brain herniation and so tranexamic acid is unlikely to improve the outcome in these patients. We therefore prespecified a sensitivity analysis that excluded these patients. However, patients with unilateral unreactive pupils were not excluded and because many of these patients have brain herniation their inclusion might also have diluted the treatment effect. Indeed, when patients with a GCS score of 3 and those with unilateral or bilateral unreactive pupils before treatment were excluded in a post-hoc analysis, the treatment effect was noticeably larger.

The effect of tranexamic acid on head injury-related death appears to depend on the time interval between injury and the initiation of the trial treatment and on the severity of TBI. Early treatment of patients with mild (GCS 13–15 and intracranial bleeding on baseline CT scan) and moderate head injury seemed to confer the greatest mortality benefit. This finding is consistent with the hypothesis that tranexamic acid improves outcome by reducing intracranial bleeding. Because haemorrhage expansion occurs in the hours immediately after injury, treatment delay would reduce the potential for tranexamic acid to prevent intracranial bleeding.^3^, ^4^ Patients with severe head injury might have less to gain from tranexamic acid treatment than patients with mild-to-moderate head injury because such patients already have extensive intracranial haemorrhage before treatment or other potentially life-threatening intracranial pathologies that are not affected by tranexamic acid. We anticipated in our statistical analysis plan that the effect of tranexamic acid would be greater for head injury-related deaths occurring in the first few days after injury than for late head injury-related deaths because early head injury-related deaths are more likely due to bleeding. Our data supports this hypothesis, showing a substantial reduction in head injury-related deaths within 24 h of injury. Similar results were obtained in the CRASH-2 trial of tranexamic acid in patients with traumatic extracranial bleeding, in which the effect of tranexamic acid on death from bleeding was greatest on the day of the injury (RR 0·72 [95% CI 0·60–0·86]).^17^ However, thereafter the benefit of tranexamic acid in head injury patients was slightly attenuated, probably because patients succumbed to non-bleeding-related pathophysiological mechanisms. This finding might explain why the effect of early tranexamic acid treatment on head injury-related death is slightly smaller than the effect of tranexamic acid on death due to bleeding seen in the CRASH-2 trial.

We found no evidence of any increased risk of adverse events. In particular, the risk of deep vein thrombosis, pulmonary embolism, stroke, and myocardial infarction was similar in the tranexamic acid and placebo groups. This finding is consistent with the results of the CRASH-2 trial, which also found no increased risk of vascular occlusive events with tranexamic acid. Unlike in the CRASH-2 trial, we found no evidence that administration beyond 3 h of injury increased the risk of head injury-related death or any other adverse events. Indeed, given the absence of any adverse effects in this trial, the implications of wrongly concluding that tranexamic acid is ineffective are likely to be far more consequential than are those of wrongly concluding that tranexamic acid is effective.

On the basis of the CRASH-2 trial results, tranexamic acid was included in guidelines for the pre-hospital care of patients with trauma. However, patients with isolated TBI were specifically excluded. The CRASH-3 trial provides evidence that tranexamic acid is safe in patients with TBI and that treatment within 3 h of injury reduces head injury-related deaths.

Correspondence to: Clinical Trials Unit, London School of Hygiene & Tropical Medicine, London WC1 E7HT, UK **crash@lshtm.ac.uk**

For **freeBIRD** see http://freebird.Lshtm.ac.uk

For the **trial protocol** see http://www.txacentral.org/

## Data sharing

Following publication of the primary and secondary analyses detailed in this statistical analysis plan, individual de-identified patient data, including a data dictionary, will be made available via our data sharing portal, The Free Bank of Injury and Emergency Research Data (freeBIRD) website indefinitely, which will allow for maximum utilisation of the data to improve patient care and advance medical knowledge. The trial protocol, statistical analysis plan, and trial publications will be freely available online.

## Supplementary Materials

Arabic translation of the abstract

Chinese translation of the abstract

French translation of the abstract

Hindi translation of the abstract

Japanese translation of the abstract

Spanish translation of the abstract

Urdu translation of the abstract
