# Department of Error

**DOI:** 10.1016/S0140-6736(19)32641-8

**Published:** 2019-11-09

*The CRASH-3 trial collaborators. Effects of tranexamic acid on death, disability, vascular occlusive events and other morbidities in patients with acute traumatic brain injury (CRASH-3): a randomised, placebo-controlled trial.* Lancet *2019; **394:** 1713–23*—In this Article, the Japanese translation of the abstract (appendix 5) has been resupplied. This correction has been made as of Nov 7, 2019.

