# Supplementary material for: Effects of tranexamic acid on death, disability, vascular occlusive events and other morbidities in patients with acute traumatic brain injury (CRASH-3): a randomised, placebo-controlled trial
Source: Lancet. 2019 Nov 9;394(10210):1713–23. doi: 10.1016/S0140-6736(19)32233-0 (PMC6853170; doi:10.1016/S0140-6736(19)32233-0)
Supplement: Arabic translation of the abstract [file mmc1.pdf]

THE LANCET

Supplementary appendix 1

This translation in Arabic was submitted by the authors and we reproduce it as supplied. It has not been peer reviewed. *The Lancet*'s editorial processes have only been applied to the original in English, which should serve as reference for this manuscript.

Supplement to: The CRASH-3 trial collaborators. Effects of tranexamic acid on death, disability, vascular occlusive events and other morbidities in patients with acute traumatic brain injury (CRASH-3): a randomised, placebo-controlled trial. *Lancet* 2019; published online Oct 14. http://dx.doi.org/10.1016/S0140-6736(19)32233-0.

تم تقديم هذه الترجمة باللغة العربية من قبل المؤلفين ونعيد إنتاجها كما هو مُقدم. إنها لم تخضع لاستعراض الأقران. تم تطبيق عمليات تحرير *لانسيت* فقط على النص الأصلي باللغة الإنجليزية، والذي يجب أن يكون بمثابة مرجع لهذه المخطوطة.

آثار حمض الترانيكساميك على الوفاة و الاعاقة ، وظواهر انسداد الأوعية الدموية وغيرها من الحالات المرضية بالمرضى الذين يعانون من إصابات الدماغ (CRASH-3): تجربة عشوائية، تستخدم دواء بديل.

تم تقديم هذه الترجمة باللغة العربية من قبل المؤلفين ونعيد إنتاجها كما هو مُقدم. إنها لم تخضع لاستعراض الأقران. تم تطبيق عمليات تحرير *لانسيت* فقط على النص الأصلي باللغة الإنجليزية، والذي يجب أن يكون بمثابة مرجع لهذه المخطوطة.

آثار حمض الترانيكساميك على الوفاة و الاعاقة ، وظواهر انسداد الأوعية الدموية وغيرها من الحالات المرضية بالمرضى الذين يعانون من إصابات الدماغ (CRASH-3): تجربة عشوائية، تستخدم دواء بديل.

معاونو التجربة CRASH-3

ملخص

مرجع: حمض الترانيكساميك يقلل من النزيف الجراحي ويخفض معدل الوفيات في المرضى الذين يعانون من نزيف خارج الجمجمة. النزيف داخل الجمجمة شائع بعد الإصابة بالدماغ (TBI) ويمكن أن يسبب فتق الدماغ والموت. قمنا بتقييم آثار حمض الترانيكساميك في مرضى TBI إصابة الدماغ.

المناهج: أُجريت هذه التجربة العشوائية في 175 مستشفى في 29 بلد. تم تعيين أول مريض في يوليو 2012 وآخر مريض في يناير 2019. البالغون المصابون بإصابة بالدماغ TBI الذين كانوا في غضون 3 ساعات من الإصابة وسجلوا 12≥ (GCS) مقياس غلاسكو للغيبوبة أو عانوا من أي نزيف داخل الجمجمة قد ظهر عبر فحص الأشعة المقطعية و لم يظهر نزيف ملحوظ خارج الجمجمة. الإطار الزمني للأهلية 8 ساعات في الأصل ولكن في عام 2016 تم تغيير البروتوكول للحد من التعيين لمن هم في غضون 3 ساعات من الإصابة. وقد أُغفل هذا التغيير عن بيانات التجربة، استجابة للأدلة الخارجية التي تفيد بأن العلاج المتأخر من غير المحتمل أن يكون فعالاً. قمنا بتعيين المرضى عشوائياً لتلقي حمض الترانيكساميك (بتحميل جرعة 1 غرام على مدار 10 دقائق ثم تسريب وريدي 1 غرام على مدار 8 ساعات) أو علاج بديل مطابق. تم تعيين المرضى عن طريق اختيار علبة علاج مُرقمة من صندوق يحتوي على ثمانية علب والتي كانت متطابقة بصرف النظر عن رقم العلبة. المرضى ، ومقدمي الرعاية ، وأولئك الذين يقومون بتقييم النتائج ، كانوا مُقنّعين للتعيين. النتيجة الأولية كانت موت نتيجة إصابة بالدماغ في المستشفى في غضون 28 يوما من الإصابة في المرضى الذين عُولجوا في غضون 3 ساعات من الإصابة. النتائج الثانوية كانت وفيات إصابة بالدماغ مبكرة، وفيات جميع الأسباب و وفيات الأسباب المحددة الإعاقة وظواهر انسداد الأوعية الدموية ، والنوبات ، والمضاعفات ، والأعراض السلبية. وقد حددنا مسبقاً تحليلاً للحساسية استبعد المرضى الذين سجلوا 3 GCS مقياس غلاسكو للغيبوبة 3 ومن لديهم عدم استجابة مزدوج لبؤبؤ العين عند القيمة القاعدية. جميع التحليلات كانت تهدف إلى المعالجة. سُجلت هذه التجربة عبر ISRCTN15088122 (19 تموز / يوليه عام 2011) ، التجارب الإكلينيكية.الحكومية رقم NCT01402882)26 يوليو 2011),EudraCT 2011-003669-14 (12 حزيران / يونيه 2012) ، سجل الدول الافريقية التجارب الإكلينيكية PACTR20121000441277 (30 تشرين الأول / أكتوبر 2012).

النتائج: بين يوليو / تموز 2012 وكانون الثاني / يناير 2019 ،قمنا بشكل عشوائي بتعيين 12,737 مريض يعانون من إصابة بالدماغ TBI لتلقي حمض الترانيكساميك أو بديله ، منهم 9,202 مريض تم معالجتهم في غضون 3 ساعات من الإصابة. من بين المرضى الذين تم معالجتهم مبكراً، خطر الموت بإصابة بالدماغ كان بنسبة 18·5% في مجموعة حمض الترانيكساميك مقابل نسبة 19·8% في مجموعة البديل (855 مقابل 892 حدث، الخطر النسبي = 0·94, 95% **CI** 0.86-1.02) في تحليل الحساسية المُحدد مسبقاً الذي استبعد المرضى الذين سجلوا 3 GCS أو من لديهم عدم استجابة مزدوج لبؤبؤ العين عند القيمة القاعدية، كانت النتائج 12·5% مجموعة حمض الترانيكساميك مقابل 14·0% مجموعة البديل (485 مقابل 525 حدث، الخطر النسبي = 0·89, 95% CI 0.80- 1.00). كان هناك انخفاض في خطر الموت بإصابة الرأس مع إستخدام حمض الترانيكساميك في حالة الإصابة الخفيفة إلى المتوسطة في الرأس (RR=0·78 95%CI 0·64-0.95) ولكن في الإصابة الخطيرة بالرأس (RR=0.99, 95%CI 0.91-1.07) لم يكن هناك أي دليل واضح على انخفاض ما (القيمة الاحتمالية للتغاير 0·030). العلاج المبكر كان أكثر فعالية في الإصابة الخفيفة والمتوسطة بالرأس (p=0·005) ولكن لم يكن هناك تأثير واضح للوقت في العلاج في الإصابة الخطيرة بالرأس (p=0·73). خطر الإعاقة وظواهر انسداد الأوعية الدموية والنوبات كان متماثلاً في كلتا المجموعتين. لم يكن هناك منفعة أو ضرر ظاهر بين أولئك الذين عشوائياً قد تجاوزوا 3 ساعات من الإصابة.

التفسير: هذه التجربة تقدم دليلاً على أن حمض الترانيكساميك آمن في حالات مرضي TBI وأن العلاج في غضون ثلاث ساعات من الإصابة يقلل من معدل وفيات إصابات الدماغ. ينبغي معالجة المرضى في أقرب وقت ممكن بعد الإصابة.

التمويل: صندوق جمعية JP الخيرية، المعهد القومي للبحوث الصحية، تجارب الصحة العالمية المشتركة (مجلس البحوث الطبية، إدارة التنمية الدولية، صندوق Wellcome)
