# Supplementary material for: Effects of tranexamic acid on death, disability, vascular occlusive events and other morbidities in patients with acute traumatic brain injury (CRASH-3): a randomised, placebo-controlled trial
Source: Lancet. 2019 Nov 9;394(10210):1713–23. doi: 10.1016/S0140-6736(19)32233-0 (PMC6853170; doi:10.1016/S0140-6736(19)32233-0)
Supplement: Chinese translation of the abstract [file mmc2.pdf]

THE LANCET

## Supplementary appendix 2

This translation in Chinese was submitted by the authors and we reproduce it as supplied. It has not been peer reviewed. *The Lancet*'s editorial processes have only been applied to the original in English, which should serve as reference for this manuscript.

Supplement to: The CRASH-3 trial collaborators. Effects of tranexamic acid on death, disability, vascular occlusive events and other morbidities in patients with acute traumatic brain injury (CRASH-3): a randomised, placebo-controlled trial. *Lancet* 2019; published online Oct 14. http://dx.doi.org/10.1016/S0140-6736(19)32233-0.

**此简体中文译文由作者提交，我方按照提供的版本刊登。此译文并未经过同行审阅。医学期刊《柳叶刀》的编辑流程仅适用于英文原稿，英文原稿应作为此手稿的参考。**

**氨甲环酸对急性创伤性颅脑损伤患者死亡、残疾、血管闭塞事件和其他发病率的影响（CRASH-3）：一项随机、安慰剂对照试验。**

**此简体中文译文由作者提交，我方按照提供的版本刊登。此译文并未经过同行审阅。医学期刊《柳叶刀》的编辑流程仅适用于英文原稿，英文原稿应作为此手稿的参考。**

# 氨甲环酸对急性创伤性颅脑损伤患者死亡、残疾、血管闭塞事件和其他发病率的影响（CRASH-3）：一项随机、安慰剂对照试验。

**CRASH-3 试验合作方**

**摘要**

*背景:* 氨甲环酸能减少创伤性颅外出血患者的手术出血，降低死亡率。颅内出血是创伤性颅脑损伤(TBI)的常见并发症，可引起脑疝和死亡。我们评估了氨甲环酸对创伤性颅脑损伤患者的作用。

*方法:* 这项随机试验在29个国家的175家医院进行。第一名患者于2012年7月招募，最后一名患者于2019年1月招募。患者符合以下条件：创伤性颅脑损伤成年患者，受伤后3小时内，格拉斯哥昏迷评分(GCS) ≤12分，或CT检查显示颅内出血且无明显颅外出血。入选的时间标准原先为8小时，但在2016年，更改了试验方案，将入选标准缩短至受伤后3小时之内，我们在未对试验数据进行分析的前提下进行了此项更改，因为外部证据表明，延迟治疗收效甚微。我们将患者随机分为两组，一组接受氨甲环酸（10分钟内负荷剂量1克，接着8小时内输注1克），另一组接受安慰剂。患者通过从盒子中选择带编号的治疗包，随机分配，盒子包含八个治疗包，除了编号，其余均一模一样。患者、护理人员和结果评估人员对分配情况均处于盲态。主要结局：受伤后3小时内入院接受治疗的患者，受伤后28天内颅脑损伤死亡；次要结局：早期颅脑损伤死亡，全因和特定原因死亡率、残疾、血管闭塞事件、癫痫发作、并发症和不良事件。我们预先规定了敏感性分析，排除了GCS评分为3分的患者以及基线期双侧瞳孔无反应的患者。所有分析均为有意治疗。本试验注册编号为ISRCTN15088122（2011年7月19日），临床试验政府编号为NCT01402882（2011年7月26日），欧洲临床试验注册中心(EudraCT)编号为2011-003669-14（2012年6月12日），泛非洲临床试验注册中心(PACTR)编号为20121000441277（2012年10月30日）。

*结果:*在2012年7月至2019年1月期间，我们将12737名TBI患者随机分为两组，一组接受氨甲环酸，另一组接受安慰剂，其中9202名患者在受伤后3小时内接受治疗。在接受早期治疗的患者中，氨甲环酸组颅脑损伤死亡的风险为18.5%，安慰剂组为19.8%（855起事件对比892起事件，相对危险为0.94, 95 % CI 0.86-1.02）。在预先规定的敏感性分析中（该分析排除了GCS评分为3分的患者或基线期双侧瞳孔无反应的患者），结果为氨甲环酸组12.5%对比安慰剂组14.0%（485起事件对比525起事件，相对危险度为0.89, 95% CI 0.80-1.00）。对于轻中度颅脑损伤(RR=0.78 95%CI 0.64-0.95)，氨甲环酸能有效降低颅脑损伤死亡风险，但对于重度颅脑损伤(RR=0.99, 95%CI 0.91-1.07)，无明显证据显示能降低风险(异质性p值0.030)。早期治疗对于轻中度颅脑损伤效果更为明显（p = 0.005），但对于重度颅脑损伤，治疗时间对于治疗效果并无明显影响（P = 0.73）。两组患者在残疾、血管闭塞事件和癫痫发作方面的风险相似。在受伤后3小时以上接受治疗的随机分组中，未显示明显的益处或危害。

*分析:* 这项试验提供了相关证据，证明氨甲环酸对于TBI患者是安全的，并且在受伤后三小时内接受治疗，能降低颅脑损伤死亡率。患者应在受伤后尽快接受治疗。

*资助方:* JP Moulton 慈善基金会、国家健康研究所、全球健康联合试验中心（惠康基金会国际发展部医疗研究委员会）。
