# Supplementary material for: Effects of tranexamic acid on death, disability, vascular occlusive events and other morbidities in patients with acute traumatic brain injury (CRASH-3): a randomised, placebo-controlled trial
Source: Lancet. 2019 Nov 9;394(10210):1713–23. doi: 10.1016/S0140-6736(19)32233-0 (PMC6853170; doi:10.1016/S0140-6736(19)32233-0)
Supplement: French translation of the abstract [file mmc3.pdf]

THE LANCET

Supplementary appendix 3

This translation in French was submitted by the authors and we reproduce it as supplied. It has not been peer reviewed. *The Lancet*'s editorial processes have only been applied to the original in English, which should serve as reference for this manuscript.

Supplement to: The CRASH-3 trial collaborators. Effects of tranexamic acid on death, disability, vascular occlusive events and other morbidities in patients with acute traumatic brain injury (CRASH-3): a randomised, placebo-controlled trial. *Lancet* 2019; published online Oct 14. http://dx.doi.org/10.1016/S0140-6736(19)32233-0.

Cette traduction en français a été proposée par les auteurs et nous l'avons reproduite telle quelle. Elle n'a pas été examinée par des pairs. Les processus éditoriaux de *Lancet* n'ont été appliqués qu'à l'original en anglais, ce qui devrait servir de référence à ce manuscrit.

Les effets de l'acide tranexamique sur la mortalité, l'invalidité, les événements vasculaires occlusifs et d'autres morbidités chez les patients atteints d'un traumatisme cranio-cérébral aigu (CRASH-3) : un essai randomisé et contrôlé par placebo.

Cette traduction en français a été proposée par les auteurs et nous l'avons reproduite telle quelle. Elle n'a pas été examinée par des pairs. Les processus éditoriaux de *Lancet* n'ont été appliqués qu'à l'original en anglais, ce qui devrait servir de référence à ce manuscrit.

Les effets de l'acide tranexamique sur la mortalité, l'invalidité, les événements vasculaires occlusifs et d'autres morbidités chez les patients atteints d'un traumatisme cranio-cérébral aigu (CRASH-3) : un essai randomisé et contrôlé par placebo.

Les collaborateurs de l'essai CRASH-3

Résumé

Contexte : L'acide tranexamique réduit les saignements chirurgicaux et diminue la mortalité chez les patients présentant des saignements extra-crâniens traumatiques. Les saignements intracrâniens sont fréquents après un traumatisme cranio-cérébral (TCC) et peuvent entraîner une hernie cérébrale et la mort. Nous avons évalué les effets de l'acide tranexamique chez les patients ayant subi un TCC.

Méthodes : Cet essai randomisé a été réalisé dans 175 hôpitaux dans 29 pays. Le premier patient a été sélectionné en juillet 2012 et le dernier en janvier 2019. Les patients admissibles étaient des adultes atteints d'un TCC dans les trois heures suivant la lésion et qui présentaient un score ≤12 sur l'échelle de coma Glasgow (GCS) ou tout saignement intracrânien à la TDM et aucun saignement extra-crânien significatif. Au départ, la période d'admissibilité était de 8 heures, mais en 2016, le protocole a été modifié pour limiter ce délai à 3 heures après une blessure. Ce changement ne permettait pas de connaitre les données de l'essai, en raison de preuves externes selon lesquelles il est peu probable qu'un traitement différé soit efficace. Nous avons randomisé les patients pour qu'ils reçoivent de l'acide tranexamique (dose de charge de 1 g sur 10 min puis perfusion de 1 g sur 8 h) ou un placebo correspondant. Les patients ont reçu un kit de traitement numéroté provenant d'une boîte contenant huit kits identiques, dont seul le numéro était différent. L'essai s'est déroulé en triple aveugle (patients, soignants et chercheurs). Le principal résultat a été le décès suite à un traumatisme crânien à l'hôpital dans les 28 jours suivant la lésion chez les patients traités dans les trois heures suivant la blessure. Parmi les résultats secondaires, mentionnons les décès par traumatisme crânien précoce, les décès pour toute cause et la mortalité par cause, l'invalidité, les événements vasculaires occlusifs, les crises, les complications et les événements indésirables. Nous avons préspécifié une analyse de sensibilité qui excluait les patients ayant un score GCS de 3 et ceux dont les pupilles bilatérales n'étaient pas réactives au départ. Toutes les analyses ont été faites dans l'intention de traiter le patient. Cet essai a été enregistré auprès de ISRCTN15088122 (19 juillet 2011), ClinicalTrials.gov numéro NCT01402882 (26 juillet 2011), EudraCT 2011-003669-14 (12 juin 2012) et le Pan African Clinical Trial Registry PACTR20121000441277 (30 octobre 2012).

Résultats : Entre juillet 2012 et janvier 2019, nous avons randomisé 12 737 patients atteints de TCC chez qui nous avons administré de l'acide tranexamique ou un placebo, dont 9 202 patients ont été traités dans les 3 heures suivant la lésion. Parmi les patients traités tôt, le risque de décès par traumatisme crânien était de 18,5 % dans le groupe traité avec de l'acide tranexamique contre 19,8 % dans le groupe placebo (855 contre 892 événements, risque relatif = 0,94, IC 95 % 0,86-1,02). Dans l'analyse de sensibilité préspécifiée qui excluait les patients ayant un score GCS de 3 ou des pupilles non réactives bilatérales au départ, les résultats étaient de 12,5 % dans le groupe sous acide tranexamique et de 14,0 % dans le groupe placebo (485 contre 525 événements, risque relatif = 0,89, IC 95 % 0,80-1,00). On a observé une réduction du risque de décès par traumatisme crânien lié à l'acide tranexamique dans les traumatismes crâniens légers à modérés (RR = 0,78 95 % CI 0,64-0,95), mais dans les traumatismes crâniens graves (RR = 0,99, 95 % CI 0,91-1,07), il n'y avait aucune preuve claire de réduction (valeur p pour hétérogénéité 0,030). Le traitement précoce était plus efficace pour les traumatismes crâniens légers et modérés (p = 0,005), mais il n'y avait pas d'impact évident du temps de traitement pour les traumatismes crâniens graves (p = 0,73). Le risque d'invalidité, d'événements vasculaires occlusifs et de crises convulsives était semblable dans les deux groupes. Il n'y a pas eu d'effet bénéfique ou nuisible apparent parmi les personnes randomisées 3 heures après la lésion.

Interprétation : Cet essai montre que l'acide tranexamique est sans danger pour les patients ayant subi un traumatisme crânien et que le traitement dans les trois heures suivant la blessure réduit le nombre de décès par traumatisme crânien. Les patients doivent être traités dès que possible après une lésion.

Financement : JP Moulton Charitable Trust, National Institute for Health Research, Joint Global Health Trials (Medical Research Council, Department for International Development, Wellcome Trust).
