# Supplementary material for: Effects of tranexamic acid on death, disability, vascular occlusive events and other morbidities in patients with acute traumatic brain injury (CRASH-3): a randomised, placebo-controlled trial
Source: Lancet. 2019 Nov 9;394(10210):1713–23. doi: 10.1016/S0140-6736(19)32233-0 (PMC6853170; doi:10.1016/S0140-6736(19)32233-0)
Supplement: Hindi translation of the abstract [file mmc4.pdf]

THE LANCET

Supplementary appendix 4

This translation in Hindi was submitted by the authors and we reproduce it as supplied. It has not been peer reviewed. *The Lancet*'s editorial processes have only been applied to the original in English, which should serve as reference for this manuscript.

Supplement to: The CRASH-3 trial collaborators. Effects of tranexamic acid on death, disability, vascular occlusive events and other morbidities in patients with acute traumatic brain injury (CRASH-3): a randomised, placebo-controlled trial. *Lancet* 2019; published online Oct 14. http://dx.doi.org/10.1016/S0140-6736(19)32233-0.

हिंदी में यह अनुवाद लेखकों द्वारा प्रस्तुत किया गया था और हम इसे जैसे उपलब्ध कराया गया वैसे पुन: पेश करते हैं। इस पर सहकर्मी की समीक्षा नहीं की गई है। लैंसेट की संपादकीय प्रक्रियाओं को केवल अंग्रेजी में मूल पर लागू किया गया है, जो इस पांडुलिपि के संदर्भ के रूप में काम आना चाहिए।

तीव्र दर्दनाक मस्तिष्क की चोट (CRASH-3) वाले रोगियों में मृत्यु, विकलांगता, संवहनी पूर्णावरोधक घटनाओं और अन्य रुग्णता पर ट्रानेक्सामिक एसिड का प्रभाव: एक यादृच्छिक, प्लेसिबो-नियंत्रित परीक्षण।

हिंदी में यह अनुवाद लेखकों द्वारा प्रस्तुत किया गया था और हम इसे जैसे उपलब्ध कराया गया वैसे पुन: पेश करते हैं। इस पर सहकर्मी की समीक्षा नहीं की गई है। लैंसेट की संपादकीय प्रक्रियाओं को केवल अंग्रेजी में मूल पर लागू किया गया है, जो इस पांडुलिपि के संदर्भ के रूप में काम आना चाहिए।

तीव्र दर्दनाक मस्तिष्क की चोट (CRASH-3) वाले रोगियों में मृत्यु, विकलांगता, संवहनी पूर्णावरोधक घटनाओं और अन्य रुग्णता पर ट्रानेक्सामिक एसिड का प्रभाव: एक यादृच्छिक, प्लेसिबो-नियंत्रित परीक्षण।

CRASH-3 परीक्षण सहयोगी

सारांश

पृष्ठभूमि: ट्रानेक्सामिक एसिड सर्जिकल रक्तस्राव को कम करता है और दर्दनाक करोटीय बाह्य रक्तस्राव वाले रोगियों में मृत्यु दर कम करता है। दर्दनाक मस्तिष्क की चोट (TBI) के बाद अन्तःकरोटीय रक्तस्राव आम है और इसके कारण मस्तिष्क की हर्नियेशन और मौत हो सकता है। हमने TBI रोगियों में ट्रानेक्सामिक एसिड के प्रभावों का आकलन किया।

प्रणाली: यह यादृच्छिक परीक्षण 29 देशों के 175 अस्पतालों में किया गया था। पहला रोगि जुलाई 2012 में और आखिरी जनवरी 2019 में भर्ती किया गया था। TBI वाले वयस्क व्यक्ति जो चोट के 3 घंटे की भीतर थे और जिनके ग्लासगो कोमा स्केल स्कोर (GCS) ≤12 था या जिनके सीटी स्कैन पर कोई अन्तःकरोटीय रक्तस्राव था और साथ ही कोई महत्वपूर्ण करोटीय बाह्य रक्तस्राव नहीं था, वे योग्य थे। योग्यता के लिए समय खिड़की मूल रूप से 8 घंटे थी, लेकिन 2016 में, चोट के 3 घंटे की भीतर तक भर्ती को सीमित करने के लिए प्रोटोकॉल को परिवर्तन किया गया था। बाहरी सबूतों के प्रतिक्रिया में कि विलंबित उपचार के प्रभावी होने की संभावना नहीं होते है, यह परिवर्तन परीक्षण के आंकड़ों को अनदेखा करके किया गया था। हमने यादृच्छिक रूप से रोगियों को ट्रानेक्सामिक एसिड (लोडिंग खुराक 1 g 10 मिनट से, फिर 1 g का आसव 8 घंटे से) या मिलान प्लेसिबो प्राप्त करने के लिए नियत किया था। पैक नंबर के अलावा सदृश थे ऐसे आठ पैक वाले एक बॉक्स से नंबरयुक्त उपचार पैक का चयन करने के माध्यम से रोगियों को नियत किया गया था। रोगियों, देखभाल करने वाले और परिणामों का आकलन करने वाले लोगों को आवंटन के लिए मुखौटा लगाया गया था। मुख्य परिणाम थे 3 घंटे के भीतर उपचार किया गया रोगियों में चोट लगने के 28 घंटे के भीतर सिर की चोट से अस्पताल में मृत्यु। गौण परिणाम थे शीघ्र सिर की चोट से मृत्यु, सभी कारण और कारण विशिष्ट मृत्यु दर, विकलांगता, संवहनी पूर्णावरोधक घटना, दौरे, जटिलता और प्रतिकूल घटनाएं। हमने एक

संवेदनशीलता विश्लेषण को पूर्व-निर्दिष्ट किया था जिसमें 3 के GCS स्कोर वाले और बेसलाइन पर द्विपक्षीय प्रतिक्रियाहीन पुतली वाले रोगियों शामिल नहीं थे। सभी विश्लेषण उपचार के इरादे से किए गए थे। यह परीक्षण ISRCTN15088122 (19 जुलाई 2011), ClinicalTrials.gov नंबर NCT01402882 (26 जुलाई 2011), EudraCT 2011-003669-14 (12 जून 2012) और पैन अफ्रीकी क्लिनिकल ट्रायल रजिस्ट्री PACTR20121000441277 (30 अक्टूबर 2012) के साथ पंजीकृत किया गया था।

परिणाम: जुलाई 2012 और जनवरी 2019 के बीच, हमने ट्रानेक्सामिक एसिड या प्लेसिबो प्राप्त करने के लिए TBI वाले 12,737 रोगियों को यादृच्छिक ढंग से आवंटित किया, जिनमें से 9,202 रोगियों का उपचार चोट के 3 घंटे की भीतर किया गया था। जल्दी उपचार किये जाने वाले रोगियों में ट्रानेक्सामिक एसिड समूह में सिर की चोट से मृत्यु का जोखिम 18·5% था बनाम 19·8% प्लेसिबो समूह में (855 बनाम 892 घटनाएँ, सापेक्ष जोखिम = 0·94, 95% सीआई 0·86- 1· 02)। पूर्व-निर्दिष्ट संवेदनशीलता विश्लेषण में जिसमें 3 के जीसीएस स्कोर वाले और बेसलाइन पर द्विपक्षीय प्रतिक्रियाहीन पुतली वाले रोगियों शामिल नहीं थे, परिणाम थे 12.5% ट्रानेक्सामिक एसिड समूह बनाम 14.0% प्लेसिबो समूह (485 बनाम 525 घटनाएँ, सापेक्ष जोखिम = 0·89, 95% सीआई 0·80- 1· 00)। हल्के से मध्यम सिर की चोट में ट्रानेक्सामिक एसिड के साथ सिर की चोट से मृत्यु के जोखिम में कमी आई थी (RR=0·78 95% CI 0·64-0·95) लेकिन गंभीर सिर की चोट में (RR= 0·99, 95% CI 0·91-1 ·07) में कमी का कोई स्पष्ट प्रमाण नहीं था (विषमता के लिए p-मान 0·030)। जल्दी उपचार हल्के और मध्यम सिर की चोट (p=0·005) में अधिक प्रभावी था, लेकिन गंभीर सिर की चोट (p=0·73) में उपचार के ऊपर समय का कोई स्पष्ट प्रभाव नहीं था। विकलांगता, संवहनी पूर्णावरोधक घटनाओं और दौरे पड़ने का जोखिम दोनों समूहों में समान था। चोट के 3 घंटे से परे उन यादृच्छिक लोगों में कोई स्पष्ट लाभ या हानि नहीं थी।

व्याख्या: यह परीक्षण इस बात का सबूत प्रदान करता है कि TBI रोगियों में ट्रानेक्सामिक एसिड सुरक्षित है और चोट के तीन घंटे के भीतर उपचार से सिर की चोटों से होने वाली मृत्यु में कमी आती है। चोट लगने पर जल्द से जल्द रोगियों का उपचार किया जाना चाहिए।

वित्त पोषण: जेपी मौलटन चैरिटेबल ट्रस्ट, राष्ट्रीय स्वास्थ्य अनुसंधान संस्थान, संयुक्त वैश्विक स्वास्थ्य परीक्षण (चिकित्सा अनुसंधान परिषद, अंतर्राष्ट्रीय विकास विभाग, वेलकम ट्रस्ट)।
