# Supplementary material for: Effects of tranexamic acid on death, disability, vascular occlusive events and other morbidities in patients with acute traumatic brain injury (CRASH-3): a randomised, placebo-controlled trial
Source: Lancet. 2019 Nov 9;394(10210):1713–23. doi: 10.1016/S0140-6736(19)32233-0 (PMC6853170; doi:10.1016/S0140-6736(19)32233-0)
Supplement: Japanese translation of the abstract [file mmc5.pdf]

THE LANCET

Supplementary appendix 5

This online publication has been corrected. The corrected version first appeared at thelancet.com on November 7, 2019.

This translation in Japanese was submitted by the authors and we reproduce it as supplied. It has not been peer reviewed. *The Lancet*'s editorial processes have only been applied to the original in English, which should serve as reference for this manuscript.

Supplement to: The CRASH-3 trial collaborators. Effects of tranexamic acid on death, disability, vascular occlusive events and other morbidities in patients with acute traumatic brain injury (CRASH-3): a randomised, placebo-controlled trial. *Lancet* 2019; published online Oct 14. http://dx.doi.org/10.1016/S0140-6736(19)32233-0.

この日本語 による翻訳文書は、著者本人により提出されたものを当方で複製したものです。本訳文の内容は査読を経ておらず、『ランセット』誌による編集も、本訳文のもとになる英語原文に対してのみ行われていることをご了承ください。

急性の外傷性脳損傷患者における死亡・身体障害・血管閉塞性事象その他罹患に対するトラネキサム酸の効果（CRASH-3）：ランダム化プラセボ対照試験

この日本語 による翻訳文書は、著者本人により提出されたものを当方で複製したものです。本訳文の内容は査読を経ておらず、『ランセット』誌による編集も、本訳文のもとになる英語原文に対してのみ行われていることをご了承ください。

急性の外傷性脳損傷患者における死亡・身体障害・血管閉塞性事象その他罹患に対するトラネキサム酸の効果（CRASH-3）：ランダム化プラセボ対照試験

CRASH-3 治験協力者

要約

背景：**トラネキサム酸は、外傷性頭蓋外出血を伴う患者における術中出血を抑えるとともに死亡率を低下させる。脳内出血は、外傷性脳損傷（TBI）後にみられやすい症状であり、脳ヘルニアの原因となったり、死に至ったりする場合がある。今回、TBI患者におけるトラネキサム酸の効果を評価したので、その結果を報告する。**

*方法：*今回のランダム化試験は29ヶ国の病院175軒において実施されたものである。最初の患者のリクルートメントは2012年7月に行われ、最後の患者のリクルートメントが2019年1月に完了した。負傷後3時間以内であり、グラスゴー昏睡尺度（GCS）が12点である、又はCTスキャンにより脳内出血が認められたが著しい頭蓋外出血を伴わない成人TBI患者を、適格対象とした。当初の適格基準による時間枠は8時間であったが、2016年にリクルートメントの範囲を制限する目的で研究実施計画書に変更がなされた際に負傷後3時間以内と設定された。治療を遅らせると効果を得られる可能性が低くなるという外部所見に鑑みて、当該の変更を秘匿化したうえで試験を継続することにした。各患者をトラネキサム酸投与群（初回投与量1gを10分以上にわたって投与後、8時間にわたり1gを注入）又は対照プラセボ投与群に無作為に割り当てた。無作為割当は、治療内容の入った封筒（治療パック）に番号を割り振り、番号以外は全く同じものを8通箱に入れ、その箱から各患者に治療パックを一つ選んでもらうことにより行われた。患者および介護人ならびにアウトカム評価者には割当情報を伏せて試験を実施した。負傷後3時間以内に治療を受けた患者における一次的アウトカムは、負傷後28日間内における院内での頭部外傷であり、二次的アウトカムとしては早期頭部外傷死、全原因死亡および原因別死亡、身体障害、血管閉塞性事象、痙攣発作、その他合併症および有害事象が挙げられる。本治験では、感度分析を、GCSが3点の患者およびベースラインで瞳が両目とも未反応の患者を除外して行うようにあらかじめ指定した。全ての分析を包括解析により行った。本治験は、ISRCTN15088122（2011年7月19日）、ClinicalTrials.gov第NCT01402882号（2011年7月26日）、EudraCT 第2011-003669-14号（2012年6月12日）、および汎アフリカ臨床治験登録（Pan-African Clinical Trial Registry）第PACTR20121000441277号（2012年10月30日）として登録済みである。

*結果：*2012年7月から2019年1月の期間に、TBI患者12,737例を、トラネキサム酸投与群又はプラセボ投与群に無作為に割り当て、うち9,202例は負傷後3時間以内に治療を受けた。早期治療を受けた患者の場合、頭部外傷死のリスクは、トラネキサム酸投与群において18.5%、プラセボ投与群においては19.8%であった（855件 対 892件，相対リスク=0.94、95% CI 0.86-1.02）。GCS3点の患者およびベースラインで瞳が両目とも未反応の患者を除外して行うようにあらかじめ指定した感度分析では、トラネキサム酸投与群において12.5%、プラセボ投与群において14.0%という結果を示した（485件 対 525件、相対リスク=0.89、95% CI 0.80-1.00）。軽・中度の頭部損傷（相対リスク=0.78、95% CI 0.80-1.00）の場合はトラネキサム酸による頭部外傷死のリスク減少が認められたが、重症の頭部損傷（相対リスク=0.99、95% CI 0.91-1.07）の場合に減少を示す明らかな兆候は認められなかった（異質性のp値＝0.030）。軽・中度の頭部損傷の場合は早期治療がより効果的である（p=0.005）が、重症の頭部損傷の場合、治療を受けるまでの時間による明らかな影響は認められなかった（p= 0.73）。身体障害や血管閉塞性事象および痙攣発作のリスクについては、両投与群において同様の結果が得られた。負傷後3時間以内に無作為割当のなされた患者においては、害も益も認められなかった。

*考察：*本治験は、トラネキサム酸がTBI患者に安全であること、そして負傷後3時間以内の治療により頭部外傷死亡率を低下できることを示す証拠を提示する。患者は負傷後早急に治療を受けるべきである。

*資金支援：*JPモールトン公益信託（JP Moulton Charitable Trust）、国立衛生研究所（National Institute for Health Research）、地球衛生共同治験（Joint Global Health Trials： 英医学研究審議会、英国際開発省、ウェルカムトラスト）。
