# Supplementary material for: Effects of tranexamic acid on death, disability, vascular occlusive events and other morbidities in patients with acute traumatic brain injury (CRASH-3): a randomised, placebo-controlled trial
Source: Lancet. 2019 Nov 9;394(10210):1713–23. doi: 10.1016/S0140-6736(19)32233-0 (PMC6853170; doi:10.1016/S0140-6736(19)32233-0)
Supplement: Spanish translation of the abstract [file mmc6.pdf]

THE LANCET

## Supplementary appendix 6

This translation in Spanish was submitted by the authors and we reproduce it as supplied. It has not been peer reviewed. *The Lancet*'s editorial processes have only been applied to the original in English, which should serve as reference for this manuscript.

Supplement to: The CRASH-3 trial collaborators. Effects of tranexamic acid on death, disability, vascular occlusive events and other morbidities in patients with acute traumatic brain injury (CRASH-3): a randomised, placebo-controlled trial. *Lancet* 2019; published online Oct 14. http://dx.doi.org/10.1016/S0140-6736(19)32233-0.

Los autores nos proporcionaron esta traducción al español y la reproducimos tal como nos fue entregada. No la hemos revisado. Los procesos editoriales de *The Lancet* se han aplicado únicamente al original en inglés, que debe servir de referencia para este manuscrito.

Efectos del ácido tranexámico sobre la muerte, discapacidad, eventos vasculares oclusivos y otras morbilidades en pacientes con lesión cerebral traumática grave (CRASH-3): estudio aleatorizado, controlado con placebo.

**Los autores nos proporcionaron esta traducción al español y la reproducimos tal como nos fue entregada. No la hemos revisado. Los procesos editoriales de *The Lancet* se han aplicado únicamente al original en inglés, que debe servir de referencia para este manuscrito.**

# Efectos del ácido tranexámico sobre la muerte, discapacidad, eventos vasculares oclusivos y otras morbilidades en pacientes con lesión cerebral traumática grave (CRASH-3): estudio aleatorizado, controlado con placebo.

**Colaboradores del estudio CRASH-3**

**Resumen**

*Antecedentes:* El ácido tranexámico reduce el sangrado por cirugía y reduce la mortalidad en pacientes con hemorragia traumática extracraneal. El sangrado intracraneal es común tras una lesión cerebral traumática (TBI por sus siglas en inglés). Se evaluó el efecto del ácido tranexámico en pacientes de TBI.

*Métodos:* Este estudio aleatorizado se llevó a cabo en 175 hospitales de 29 países. El primer paciente fue reclutado en julio de 2012 y, el último, en enero de 2019. Para el estudio se admitía a adultos con TBI que se encontraban dentro de las 3 primeras horas desde la lesión y que tenían una puntuación de ≤12 en la escala de coma de Glasgow (GCS por sus siglas en inglés), o cuyo TAC mostraba alguna hemorragia intracraneal sin una hemorragia extracraneal significativa. El periodo de tiempo para la selección era originalmente de 8 horas, pero en 2016 se cambió el protocolo para limitar la admisión a un periodo de 3 horas desde la lesión. Este cambio se ignoró en los datos del estudio y en respuesta a las evidencias externas de que es poco probable que un tratamiento tardío sea eficaz. De forma aleatoria, unos pacientes recibieron un tratamiento con ácido tranexámico (dosis de carga 1 g durante 10 min y luego 1 g por infusión a lo largo de 8 horas) y, otros, un tratamiento con efecto placebo. La asignación de pacientes se realizó mediante la elección de un paquete de tratamiento numerado. El paquete se encontraba dentro de una caja en la que había ocho paquetes idénticos, excepto por el número. La asignación se ocultó a pacientes, cuidadores y personas encargadas de evaluar los resultados. El resultado principal fue la muerte por traumatismo craneal en el hospital en los primeros 28 días desde la lesión en pacientes tratados dentro de las 3 primeras horas desde el traumatismo. Los resultados secundarios fueron muertes prematuras por traumatismo craneal, mortalidad en general y por causa específica, discapacidad, episodios vasculares oclusivos, convulsiones, complicaciones y eventos adversos. Se hizo una especificación de análisis previo de sensibilidad que excluyó a los pacientes con una puntuación de 3 en la escala GCS y a los que presentaban ambas pupilas no reactivas al inicio del estudio. Todos los análisis fueron por intención de tratar. Este estudio se registró en ISRCTN15088122 (19 de julio de 2011), ClinicalTrials.gov número NCT01402882 (26 de julio de 2011), EudraCT 2011-003669-14 (12 de junio de 2012) y en el Registro de Estudios Clínicos Pan Africanos PACTR20121000441277 (30 de octubre de 2012).

*Resultados:* Entre julio de 2012 y enero de 2019, se asignaron aleatoriamente 12 737 pacientes con TBI a un tratamiento con ácido tranexámico o a un placebo. A 9202 de ellos se les trató dentro de las 3 primeras horas desde la lesión. El riesgo de muerte por traumatismo craneal entre los pacientes a quienes se trató temprano fue del 18,5 % en el grupo del ácido tranexámico y del 19,8 % en el grupo del placebo (855 frente a 892 eventos, riesgo relativo = 0,94, CI (Intervalo de Confianza) de 95 % 0,86-1,02). En el análisis de sensibilidad previo especificado que excluyó a pacientes con una puntuación de 3 en la escala GCS o con ambas pupilas no reactivas al inicio del estudio, los resultados fueron de 12,5 % para el grupo del ácido tranexámico y 14,0 % para el grupo del placebo (485 frente a 525 eventos, riesgo relativo = 0,89, CI de 95 % 0,80-1,00). Hubo una reducción del riesgo de muerte por traumatismo craneal con el ácido tranexámico en los casos de traumatismo craneal leve o moderado (RR=0,78 CI de 95 % 0,64-0,95), pero en los casos de traumatismo craneal grave (RR=0,99, CI de 95 % 0,91-1,07) no hay evidencias de disminución (valor p para la heterogeneidad 0,030). El tratamiento temprano fue más eficaz en los casos de traumatismo craneal leve y moderado (p=0,005), pero el momento del tratamiento no tuvo un efecto evidente en los casos de traumatismo craneal grave (p=0,73). El riesgo de discapacidad, eventos vasculares oclusivos y convulsiones fue parecido en ambos grupos. No hubo ni beneficios ni daños aparentes entre los pacientes aleatorizados después de las 3 primeras horas desde la lesión.

*Interpretación:* Este estudio ofrece evidencias de que el ácido tranexámico es seguro para pacientes de TBI y que el tratamiento dentro de las tres primeras horas desde la lesión reduce las muertes por traumatismo craneal. Hay que tratar a los pacientes lo antes posible después de la lesión.

*Financiación:* JP Moulton Charitable Trust, National Institute for Health Research, Joint Global Health Trials (Consejo de Investigación Médica, Departamento para el Desarrollo Internacional, Wellcome Trust).
