# Supplementary material for: Effects of tranexamic acid on death, disability, vascular occlusive events and other morbidities in patients with acute traumatic brain injury (CRASH-3): a randomised, placebo-controlled trial
Source: Lancet. 2019 Nov 9;394(10210):1713–23. doi: 10.1016/S0140-6736(19)32233-0 (PMC6853170; doi:10.1016/S0140-6736(19)32233-0)
Supplement: Urdu translation of the abstract [file mmc7.pdf]

THE LANCET

Supplementary appendix 7

This translation in Urdu was submitted by the authors and we reproduce it as supplied. It has not been peer reviewed. *The Lancet*'s editorial processes have only been applied to the original in English, which should serve as reference for this manuscript.

Supplement to: The CRASH-3 trial collaborators. Effects of tranexamic acid on death, disability, vascular occlusive events and other morbidities in patients with acute traumatic brain injury (CRASH-3): a randomised, placebo-controlled trial. *Lancet* 2019; published online Oct 14. http://dx.doi.org/10.1016/S0140-6736(19)32233-0.

اردو میں یہ ترجمہ مصنفین کے ذریعہ پیش کیا گیا تھا اور ہم اس کی ہو بہو دوبارہ تخلیق کرتے ہیں۔ اس کا ہم مرتبہ ماہروں کے ذریعہ جائزہ نہیں لیا گیا ہے۔ *لانسیٹ* کے ادارتی عمل کا اطلاق صرف انگریزی کے اصل پر ہوا ہے، جو اس نسخے کے حوالہ کے طور پر کام کرے گا

شدید تکلیف دہ دماغی چوٹ کے مریضوں میں موت، معذوری، عصبی رکاوٹ کے واقعات اور دیگر امراض پر ٹرانیکسامک ایسڈ کے اثرات (CRASH-3): ایک بے ترتیب، پلیسبو کنٹرول شدہ تجربہ۔

اردو میں یہ ترجمہ مصنفین کے ذریعہ پیش کیا گیا تھا اور ہم اس کی ہو بہو دوبارہ تخلیق کرتے ہیں۔ اس کا ہم مرتبہ ماہروں کے ذریعہ جائزہ نہیں لیا گیا ہے۔ *لانسیٹ* کے ادارتی عمل کا اطلاق صرف انگریزی کے اصل پر ہوا ہے، جو اس نسخے کے حوالہ کے طور پر کام کرے گا

شدید تکلیف دہ دماغی چوٹ کے مریضوں میں موت، معذوری، عصبی رکاوٹ کے واقعات اور دیگر امراض پر ٹرانیکسامک ایسڈ کے اثرات (CRASH-3): ایک بے ترتیب، پلیسبو کنٹرول شدہ تجربہ۔

CRASH-3 تجربہ کے شراکت دار

خلاصه

پس منظر: ٹرانیکسامک ایسڈ جراحی جریان خون کو روکتا ہے اور تکلیف دہ کھوپڑی سے ماورا جریان خون والے مریضوں میں اموات کو کم کرتا ہے۔ تکلیف دہ دماغی چوٹ (TBI) کے بعد کھوپڑی کے اندر خون بہنا عام بات ہے اور یہ دماغی فتقیت اور موت کا سبب بن سکتا ہے۔ ہم نے TBI کے مریضوں میں ٹرانیکسامک ایسڈ کے اثرات کا اندازہ کیا۔

طریقہ کار: یہ بے ترتیب تجربہ 29 ممالک کے 175 اسپتالوں میں کیا گیا۔ پہلا مریض جولائی 2012 اور آخری بار جنوری 2019 میں بھرتی کیا گیا تھا۔ TBI والے بالغ افراد جنھیں 3 گھنٹوں کے اندر چوٹ لگی تھی اور جن کا گلاسگو کوما اسکیل اسکور (جی سی ایس) ≤12 تھا یا سی ٹی اسکین پر کھوپڑی کے اندر خون بہہ رہا تھا اور کوئی خاص کھوپڑی کے ماورا خون نہیں بہہ رہا تھا وہ اس کے لیے اہل تھے۔ اہلیت کے لیے وقت کی حد اصل میں 8 گھنٹے تھی لیکن 2016 میں پروٹوکول کو تبدیل کر کے بھرتی کو چوٹ کے 3 گھنٹے کے اندر محدود کر دیا گیا تھا۔ اس تبدیلی کو ان بیرونی شواہد کے جواب میں تجربے کے ڈیٹا سے مٹا دیا گیا تھا کہ تاخیر سے علاج کے مؤثر ہونے کا امکان نہیں ہے۔ ہم نے اٹکل پچو طریقے سے مریضوں کو ٹرانیکسامک ایسڈ (لوڈنگ خوراک 10 منٹ میں 1 گرام اور پھر 8 گھنٹوں میں 1 گرام انفیوزن یعنی عرق) اور مماثل پلیسبو حاصل کرنے کے لیے مختص کیا۔ مریضوں کو آٹھ پیک پر مشتمل ایک باکس سے نمبر ڈالے گیے علاج کے پیک کے انتخاب کے ذریعہ تفویض کیا گیا تھا جو پیک نمبر کے علاوہ ایک جیسے تھے۔ مریض، دیکھ بھال فراہم کرنے والے، اور نتائج کا اندازہ کرنے والے مختص کرنے سے رضامند تھے۔ بنیادی نتیجہ چوٹ کے 3 گھنٹوں کے اندر علاج کیے گئے مریضوں میں چوٹ کے 28 دنوں کے اندر ہسپتال میں سر میں چوٹ کی وجہ سے موت تھی۔ ثانوی نتائج سر کی چوٹ سے جلدی اموات، تمام سبب اور مخصوص سبب سے موت، معذوری، عروقی رکاوٹ کے واقعات، دورے، پیچیدگیاں، اور منفی واقعات تھے۔ ہم نے حساسیت کے تجزیہ کو پہلے سے طے کیا تھا جس میں 3 جی سی ایس اسکور والے اور بیس لائن میں دو طرفہ غیر فعال پتلیوں والے مریضوں کو خارج کر دیا گیا تھا۔ تمام تجزیے علاج کرنے کے ارادے سے تھے۔ یہ تجربہ ISRCTN15088122 (19 جولائی 2011)، ClinicalTrials.gov نمبر NCT01402882 (26 جولائی 2011)،EudraCT 2011-003669-14 (12 جون 2012)، اور پین افریقی کلینکل ٹرائل رجسٹری PACTR20121000441277 (30 اکتوبر 2012) کے ساتھ رجسٹر کیا گیا تھا۔

نتائج: جولائی 2012 اور جنوری 2019 کے درمیان، ہم نے اٹکل پچو طریقے سے ٹی بی آئی کے 12,737 مریضوں کو ٹرانیکسامک ایسڈ یا پلیسبو حاصل کرنے کے لیے مختص کیا، جن میں سے 9,202 مریضوں کا علاج چوٹ کے 3 گھنٹوں کے اندر کیا گیا۔ جلدی علاج کیے جانے والے مریضوں میں، سر کے چوٹ سے موت کا خطرہ ٹرانیکسامک ایسڈ گروپ میں 18·5% بمقابلہ پلیسبو گروپ میں 19·0%8 تھا (855 بمقابلہ 892 واقعات، خطرے کا تناسب = 0·94، 95% سی آئی 0·86-1·02)۔ پہلے سے طے شدہ حساسیت کے تجزیہ، جس میں 3 جی سی ایس اسکور والے یا بیس لائن میں دو طرفہ غیر فعال پتلیوں والے مریضوں کو خارج کر دیا گیا تھا، میں نتائج 12·5% ٹرانیکسامک ایسڈ گروپ بمقابلہ 14·0% پلیسبو گروپ تھے (485 بمقابلہ 525 واقعات، خطرے کا تناسب = 0·89، 95% سی آئی 0·80-1·00)۔ ہلکے سے معتدل سر کی چوٹ (آر آر = 0·78، 95% سی آئی 0·64-0·95) میں ٹرانیکسامک ایسڈ کے ساتھ سر کی چوٹ سے موت کے خطرے میں کمی ہوئی تھی لیکن سر کی شدید چوٹ (آر آر= 0·99، 95% سی آئی 0·91-1·07) میں کمی کا واضح ثبوت موجود نہیں تھا (مختلف النوعیت 0·030 کے لیے پی-ویلیو)۔ جلدی علاج ہلکی اور معتدل سر کی چوٹ (پی=0·005) میں زیادہ مؤثر تھا لیکن سر کی شدید چوٹ (پی=0·73) میں علاج کے وقت پر اس کا کوئی واضح اثر نہیں ہوا۔ معذوری، عروقی رکاوٹ کے واقعات اور دوروں کا خطرہ دونوں گروپوں میں ایک جیسا تھا۔ 3 گھنٹے سے زیادہ کی چوٹ والے اٹکل پچو ترتیب والوں میں کوئی واضح فائدہ یا نقصان نہیں تھا۔

تشریح: یہ تجربہ اس بات کا ثبوت فراہم کرتا ہے کہ ٹی بی آئی مریضوں میں ٹرانیکسامک ایسڈ محفوظ ہے اور چوٹ کے تین گھنٹوں کے اندر علاج سے سر کی چوٹ کی اموات کم ہو جاتی ہیں۔ چوٹ کے بعد جلد سے جلد مریضوں کا علاج کیا جانا چاہئے۔

فنڈنگ: جے پی مولٹن چیریٹیبل ٹرسٹ، نیشنل انسٹی ٹیوٹ برائے ہیلتھ ریسرچ، جوائنٹ گلوبل ہیلتھ ٹرائلز (میڈیکل ریسرچ کونسل، محکمہ برائے بین الاقوامی ترقی، ویلکم ٹرسٹ)۔
