# Supplementary material for: Effects of tranexamic acid on death, disability, vascular occlusive events and other morbidities in patients with acute traumatic brain injury (CRASH-3): a randomised, placebo-controlled trial
Source: Lancet. 2019 Nov 9;394(10210):1713–23. doi: 10.1016/S0140-6736(19)32233-0 (PMC6853170; doi:10.1016/S0140-6736(19)32233-0)
Supplement: Supplementary appendix [file mmc8.pdf]

THE LANCET

Supplementary appendix 8

This appendix formed part of the original submission and has been peer reviewed. We post it as supplied by the authors.

Supplement to: The CRASH-3 trial collaborators. Effects of tranexamic acid on death, disability, vascular occlusive events and other morbidities in patients with acute traumatic brain injury (CRASH-3): a randomised, placebo-controlled trial. *Lancet* 2019; published online Oct 14. http://dx.doi.org/10.1016/S0140-6736(19)32233-0.

Web appendix

Web appendix Table 1. Baseline characteristics prior to randomisation in all patients and in those randomised beyond 3 hours of injury

	**All**				**≥3 hours**			
	**TXA (n=6406)**		**Placebo (n=6331)**		**TXA (n=1757)**		**Placebo (n=1778)**	
	n	(%)	n	(%)	n	(%)	n	(%)
**Sex**								
Male	5104	(80)	5013	(79)	1362	(78)	1353	(76)
Female	1301	(20)	1318	(21)	395	(22)	425	(24)
Unknown	1	(<1)	0	(0)	0	(0)	0	(0)
**Age (years)**								
Mean (SD)	*43*	*(19.8)*	*43.1*	*(19.7)*	*46.4*	*(21.3)*	*46.2*	*(21.1)*
<24	1362.0	(21)	1326	(21)	320	(18)	330	(19)
24-44	2285	(36)	2264	(36)	569	(32)	592	(33)
44-64	1625	(25)	1613	(25)	456	(26)	429	(24)
>64	1134	(18)	1128	(18)	412	(23)	427	(24)
**Time since injury (hours)**								
Mean (SD)	*2.9*	*(3.2)*	*2.9*	*(2.3)*	*5.5*	*(5.2)*	*5.4*	*(2.9)*
≤1	877	(14)	869	(14)	-	-	-	-
1-3	3772	(59)	3684	(58)	-	-	-	-
3-8	1737	(27)	1760	(28)	1737	(99)	1760	(99)
>8	20	(<1)	18	(<1)	20	(1)	18	(1)
**Systolic blood pressure (mm Hg)**								
<90	108	(2)	109	(2)	19	(1)	24	(1)
90-119	2001	(31)	1988	(31)	493	(28)	498	(28)
120-139	2107	(33)	2120	(33)	646	(37)	616	(35)
≥140	2167	(34)	2097	(33)	591	(34)	631	(35)
Unknown	23	(<1)	17	(<1)	8	(<1)	9	(1)
**Glasgow Coma Scale (GCS)**								
3	630	(10)	642	(10)	135	(3)	136	(3)
4	261	(4)	275	(4)	48	(1)	62	(1)
5	211	(3)	242	(4)	48	(1)	70	(2)
6	304	(5)	308	(5)	83	(2)	76	(2)
7	413	(6)	400	(6)	102	(2)	106	(2)
8	465	(7)	406	(6)	111	(2)	91	(2)
9	416	(6)	382	(6)	81	(2)	90	(2)
10	463	(7)	444	(7)	92	(2)	80	(2)
11	465	(7)	502	(8)	90	(2)	112	(2)
12	600	(9)	601	(9)	124	(3)	123	(3)
13	460	(7)	453	(7)	163	(4)	141	(3)
14	790	(12)	754	(12)	264	(6)	296	(7)
15	899	(14)	886	(14)	415	(9)	394	(9)
Unknown	29	(<1)	36	(1)	1	(<1)	1	(<1)
**Pupil reaction**								
None react	536	(8)	575	(9)	111	(6)	135	(8)
One reacts	511	(8)	482	(8)	137	(8)	129	(7)
Both react	5174	(81)	5113	(81)	1468	(84)	1477	(83)
Unable to assess/unknown	185	(3)	161	(3)	41	(2)	37	(2)

SD=standard deviation; TXA=tranexamic acid

Web appendix Table 2. Effect of tranexamic acid on non-head injury deaths in all patients

Cause of death	TXA (n=6359)		Placebo (n=6280)		RR (95% CI)
	**n**	**(%)**	**n**	**(%)**	
Bleeding	9	(0.1)	7	(0.1)	1.27 (0.47-3.41)
Pulmonary embolism	9	(0.1)	7	(0.1)	1.27 (0.47-3.41)
Stroke	10	(0.2)	4	(0.1)	2.47 (0.77-7.87)
Myocardial infarction	9	(0.1)	3	(<0.1)	2.96 (0.80-10.94)
Multi-organ failure	27	(0.4)	24	(0.4)	1.11 (0.64-1.92)
Aspiration/pneumonia	30	(0.5)	34	(0.5)	0.87 (0.53-1.42)
Sepsis	9	(0.1)	6	(0.1)	1.48 (0.53-4.16)
Cervical spine injury	3	(<0.1)	4	(0.1)	0.74 (0.17-3.31)
Other	16	(0.3)	11	(0.2)	1.44 (0.67-3.09)

CI=confidence interval, RR=risk ratio, SD=standard deviation, TXA=tranexamic acid.

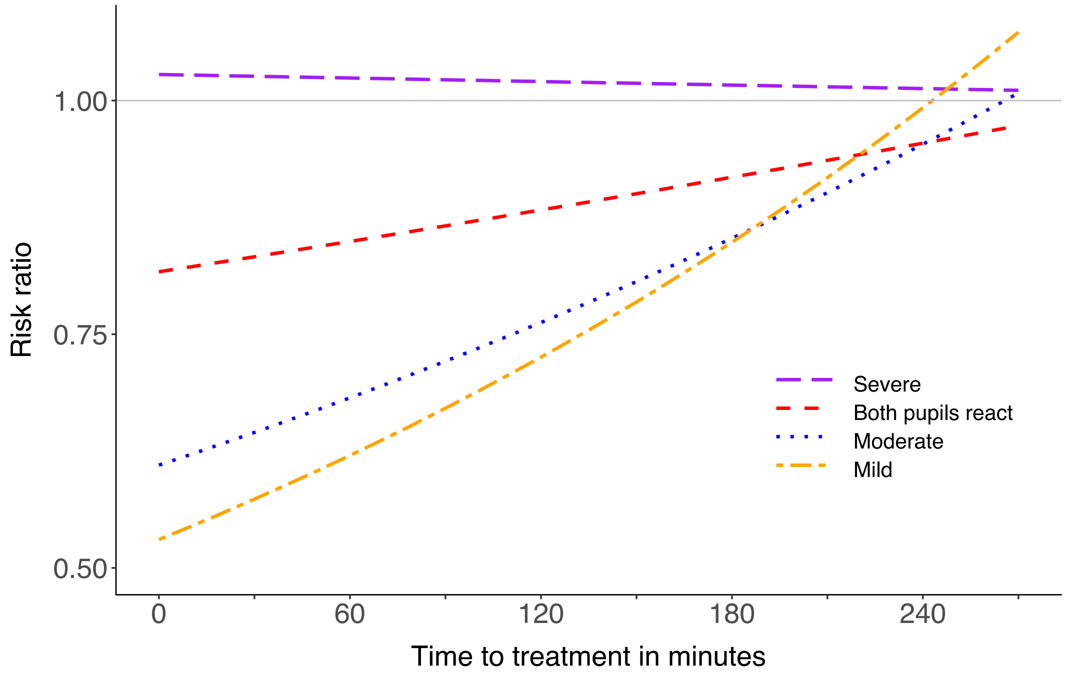

Web appendix Figure 1: Effectiveness of tranexamic acid versus time to treatment stratified by severity in all patients

Web appendix Table 3: Adverse events by MedDRA term

	TXA (N=6359)	Placebo (N=6280)	Total (N=12639)
**Any adverse event**	**198**	**168**	**366**
Pneumonia	51	50	101
Respiratory infection	10	7	17
Fall	11	5	16
Urinary tract infection	9	5	14
Abnormal liver function tests	6	6	12
Allergic reaction	4	5	9
Cellulitis	4	4	8
Wound infection	4	3	7
Atrial fibrillation	5	1	6
Headache	5	1	6
Pneumothorax	4	2	6
Supraventricular tachycardia	3	2	5
Cerebral haemorrhage	1	3	4
Ileus	1	3	4
Pyrexia	2	2	4
Urinary retention	3	1	4
Cardiac arrest	3	0	3
Chest pain	3	0	3
Constipation	1	2	3
Haemothorax	1	2	3
Heart block	1	2	3
Infection MRSA	1	2	3
Intracranial venous sinus thrombosis	1	2	3
Meningitis	1	2	3
Pulmonary embolism	3	0	3
Respiratory failure	0	3	3
Acute respiratory distress syndrome	1	1	2
Anaemia	0	2	2
Atrial flutter	1	1	2
Cerebral haematoma	2	0	2
Clostridium difficile infection	2	0	2
Diarrhoea	2	0	2
Epilepsy	0	2	2
Gangrene	2	0	2
Hypertension	0	2	2
Hypokalaemia	1	1	2
Intestinal pseudo-obstruction	1	1	2
Ischaemic stroke	2	0	2
Neutropenia	2	0	2
Pancreatitis	1	1	2
Rash	1	1	2
Respiratory arrest	1	1	2
Seizure	1	1	2
Sepsis	1	1	2
Thrombocytopenia	2	0	2
Thrombocytosis	1	1	2
Traumatic brain injury	1	1	2
Unintended unilateral bronchial intubation	1	1	2
Wound dehiscence	0	2	2
Abdominal compartment syndrome	1	0	1
Abdominal distension	0	1	1
Acute alcoholic intoxication	1	0	1
Agitation	1	0	1
Atelectasis	0	1	1
Bacteraemia	0	1	1
Bowel obstruction	1	0	1

Bradycardia	0	1	1
Central line infection	1	0	1
Cerebral salt-wasting syndrome	1	0	1
Cerebrospinal fluid leakage	0	1	1
Cerebrospinal infection	1	0	1
Cervical pain	0	1	1
Corneal ulcer	1	0	1
Cranial nerve palsies multiple	0	1	1
Cranial nerve paralysis	1	0	1
Depression	1	0	1
Diabetic ketoacidosis	1	0	1
ECG signs of myocardial ischaemia	0	1	1
Eye injury	1	0	1
Facial palsy	0	1	1
Foot drop	0	1	1
Fractured zygomatic arch reduction	0	1	1
Haematoma	1	0	1
Haematuria	0	1	1
Haemophilus influenza pneumonia	0	1	1
Herpes zoster	1	0	1
Hip dislocation	0	1	1
Humerus fracture	1	0	1
Hydrocephalus	0	1	1
Hyperbilirubinaemia	1	0	1
Hypernatraemia	1	0	1
Hyponatraemia	1	0	1
Hypotension	1	0	1
Hypothermia	0	1	1
Jaw pain	1	0	1
Laceration of head	1	0	1
Laryngopharyngitis	1	0	1
Leg pain	0	1	1
Liver failure	0	1	1
Metabolic encephalopathy	1	0	1
Necrotising fasciitis	0	1	1
Neuroleptic malignant syndrome	0	1	1
Obstructive jaundice	0	1	1
Overdose	0	1	1
Painful urination	0	1	1
Paraesthesia	1	0	1
Pleural effusion	0	1	1
Post procedural infection	0	1	1
Psychotic episode	1	0	1
Pulmonary haemorrhage	1	0	1
Pulmonary oedema	0	1	1
Rectal bleeding	1	0	1
Shunt infection	1	0	1
Sinus pause	1	0	1
Stroke	1	0	1
Thrombocythaemia	0	1	1
Thyroid haemorrhage	1	0	1
Toothache	0	1	1
Tracheostomy	0	1	1
Tracheostomy complication	0	1	1
Tracheostomy infection	1	0	1
Vasovagal reaction	0	1	1
Ventricular fibrillation	0	1	1
Ventricular tachycardia	0	1	1
Ventriculitis	1	0	1
Vocal cord paresis	1	0	1

TXA=tranexamic acid
